# A survey of functional genomic variation in domesticated chickens

**DOI:** 10.1186/s12711-018-0390-1

**Published:** 2018-04-16

**Authors:** Martijn F. L. Derks, Hendrik-Jan Megens, Mirte Bosse, Jeroen Visscher, Katrijn Peeters, Marco C. A. M. Bink, Addie Vereijken, Christian Gross, Dick de Ridder, Marcel J. T. Reinders, Martien A. M. Groenen

**Affiliations:** 1 Wageningen University & Research Animal Breeding and Genomics, P.O. Box 338, 6700 AH Wageningen, The Netherlands; 2 Hendrix Genetics Research Technology & Service B.V., P.O. Box 114, 5830 AC Boxmeer, The Netherlands; 3 Bioinformatics Group, Wageningen University and Research, P.O. Box 633, 6708 PB Wageningen, The Netherlands; 4 Delft Bioinformatics Lab, Delft University of Technology, Delft, The Netherlands

## Abstract

**Background:**

Deleterious genetic variation can increase in frequency as a result of mutations, genetic drift, and genetic hitchhiking. Although individual effects are often small, the cumulative effect of deleterious genetic variation can impact population fitness substantially. In this study, we examined the genome of commercial purebred chicken lines for deleterious and functional variations, combining genotype and whole-genome sequence data.

**Results:**

We analysed over 22,000 animals that were genotyped on a 60 K SNP chip from four purebred lines (two white egg and two brown egg layer lines) and two crossbred lines. We identified 79 haplotypes that showed a significant deficit in homozygous carriers. This deficit was assumed to stem from haplotypes that potentially harbour lethal recessive variations. To identify potentially deleterious mutations, a catalogue of over 10 million variants was derived from 250 whole-genome sequenced animals from three purebred white-egg layer lines. Out of 4219 putative deleterious variants, 152 mutations were identified that likely induce embryonic lethality in the homozygous state. Inferred deleterious variation showed evidence of purifying selection and deleterious alleles were generally overrepresented in regions of low recombination. Finally, we found evidence that mutations, which were inferred to be evolutionally intolerant, likely have positive effects in commercial chicken populations.

**Conclusions:**

We present a comprehensive genomic perspective on deleterious and functional genetic variation in egg layer breeding lines, which are under intensive selection and characterized by a small effective population size. We show that deleterious variation is subject to purifying selection and that there is a positive relationship between recombination rate and purging efficiency. In addition, multiple putative functional coding variants were discovered in selective sweep regions, which are likely under positive selection. Together, this study provides a unique molecular perspective on functional and deleterious variation in commercial egg-laying chickens, which can enhance current genomic breeding practices to lower the frequency of undesirable variants in the population.

**Electronic supplementary material:**

The online version of this article (10.1186/s12711-018-0390-1) contains supplementary material, which is available to authorized users.

## Background

In animal breeding, the number of deleterious genetic variants that are segregating in a population is affected by several factors, e.g. genetic drift, mutation rate, and selection. As a result, small effective population size and artificial selection can impact population fitness in domesticated populations substantially [1] and can lead to a high risk of inbreeding depression, which is the result of the accumulation of deleterious alleles that increase in frequency, mainly due to genetic drift [1]. Deleterious alleles are expected to be purged from the population by purifying selection, and thus, generally remain at low frequencies in a population [2]. However, many evolutionary forces shape the landscape of deleterious alleles in a population, including recombination and genetic hitchhiking, which is a change in allele frequency due to the allele being passed along together with a variant that is under selection [3]. Recent examples have shown a large impact of such deleterious alleles in several livestock populations [4, 5]. Therefore, effective purging of these deleterious variants is desired. However, most of these variants are rare, and selection on rare variants is usually inefficient, especially if the relationship between genotype and phenotype is poorly characterised [6, 7].

In this study, we examined chicken layer lines that have been primarily selected for production traits, including mortality, egg production, egg composition, shell quality [8], and traits related to animal welfare [9]. In spite of the many positive consequences of this artificial selection, several health issues are associated with intense selection for production traits in laying hens, including excessive comb growth, brittle bones, feather pecking, and ovarian cancer [10–12]. To date, the underlying genetic architecture of these deleterious effects has not been characterised. Therefore, it is essential to better understand the relationship between genotype and phenotype, which is, to a large extent, still a black box [13].

Purebred chickens are routinely genotyped by breeding companies using SNP genotyping panels to accelerate genetic progress by applying genomic selection [14]. Although genomic selection itself may not be very efficient in eliminating low-frequency deleterious variants, the large number of routinely genotyped and pedigreed individuals does allow for the identification of deleterious variation. A powerful method is to systematically assess missing homozygosity in the genome by identifying haplotypes that cause early lethality by statistical depletion, or even absence, of the homozygous state, suggesting that they carry a lethal recessive mutation [4]. This approach can detect even very rare (frequency < 2%) deleterious haplotypes if a large number, at least several thousands, of animals are genotyped in a population. One disadvantage of this method is that low-frequency deleterious variants that reside on common haplotypes will be missed [5]. An alternative method that does allow such rare deleterious alleles to be identified is to sequence the entire genome of tens to hundreds of animals from a population. Whole-genome sequencing (WGS) can be used to identify potential phenotype-altering variants, which can range from embryonic lethal (EL) to only mildly deleterious mutations in coding regions, and to predict their effects using various tools [15]. The use of WGS data from a population can lead to the discovery of variants that are beneficial for breeding programs [16, 17], e.g. by looking for regions in the genome that are under (recent) positive selection. A challenge for this approach is to differentiate true selected variants and variants that increased in frequency as a result of genetic drift. In addition, the incompleteness of current genome annotations in most livestock species hampers the identification of such variants.

In this study, we combined two complementary approaches to identify deleterious and functional variation (positively selected variants in relation to traits under selection) in purebred commercial layer lines. First, we showed that missing homozygosity can result from early embryonic lethality. Second, we mined the genomes of 250 whole-genome-sequenced individuals for deleterious (including embryonic lethal) and functional variants. The result is a comprehensive catalogue of putative deleterious and functional variants, which will be an important resource for future functional studies in chicken and should facilitate the purging of deleterious variants in breeding populations.

## Methods

### Animals, genotypes and pre-processing

We genotyped six different commercial chicken breeds using the 60 K Illumina SNP BeadChip: one purebred white layer dam line (WA), one purebred white layer sire line (W1), two crossbred lines (CB: W1-WA, W1-WD) and two brown layer lines (B1, B2) (see Additional file 1: Table S1). All animals from multiple generations were genotyped as part of a routine data collection from Hendrix-Genetics breeding programs. Chromosomal positions were determined based on the *Gallus gallus* GalGal5 reference assembly [18]. SNPs with an unknown position on the Galgal5 reference assembly and SNPs on sex chromosomes were discarded. Pre-processing was performed using PLINK v1.90b3.30 [19, 20] based on the following criteria: each SNP had to have a minor allele frequency higher than 0.01 (1%) and a call rate higher than 0.85 and animals with a call rate lower than 0.7 were discarded from the analysis. We did not filter for deviations from Hardy–Weinberg equilibrium (HWE) because haplotypes that exhibit a deficit in homozygosity were expected to deviate from HWE.

### Phasing and identification of missing homozygous haplotypes

We used the BEAGLE version 4.0 genetic analysis software for phasing of the SNP genotypes [21]. We used a sliding-window approach using window sizes ranging from 0.25 to 1 Mb in steps of 0.5 times the window size. Haplotypes with a frequency higher than 0.5% were retained for identification of missing homozygotes. The expected number of homozygotes was estimated using the parental haplotype information with the formula described by Fritz et al. [22]. The number of heterozygous offspring from carrier matings was also calculated to verify whether there was a deviation from HWE. An exact binomial test was applied to compare the number of observed versus expected homozygotes. Haplotypes were considered significantly depleted of homozygotes if the *p* value for this test was less than 0.005.

### Population sequencing and mapping

We used WGS data from three commercial white layer lines, two dam lines (WA: 71, WD: 78) and one sire line (W1: 101), and sequenced a total of 3.502 Tbp (tera base pairs) from 35.94 billion paired-end 100 bp reads sequenced on an Illumina HiSeq machine. We used Sickle software to trim the sequences [23], BWA-MEM (version 0.7.15, [24]) to map the WGS data to the chicken reference genome (Galgal5) [18], the Samtools dedup function to discard duplicate reads [25], and GATK IndelRealigner to perform local realignments of reads around indels [26].

### Variant detection and post-processing

We performed population-based variant calling using Freebayes software taking the aligned BAM files as input with the following settings: —min-base-quality 10—min-alternate-fraction 0.2—haplotype-length 0—pooled-continuous—ploidy 2—min-alternate-count 2 [27]. Post-processing was performed using bcftools [25] and variants that were located within 3 bp of an indel, or with a phred quality score and call rate below 20 and 0.7, respectively, were discarded. Moreover, genotype calls were filtered for sample depth (min: 4, max: AvgDepth * 2.5).

### Candidate gene identification

We imputed the 250 WGS animals to 60 K genotypes, to match 60 K-based haplotypes to the available sequence data. The software Confirm-gt [21] was used to match chromosome, strand, and allele to the phased 60 K reference population. BEAGLE version 4.0 was used for imputation and phasing. Carriers of haplotypes that were significantly depleted of homozygotes were examined for causal variants by selecting protein-altering variants carried uniquely by the haplotype carriers. We used the variant effect predictor (VEP, Ensembl-release 86) to predict the impact of the candidate variants identified [28]. The impact of the missense variants were assessed using the SIFT and PROVEAN software tools [29, 30].

### Population statistics

Principle component analysis was performed using PLINK on the filtered vcf files and plotted using the R package ggplot2. PLINK was used with the—het option to calculate the inbreeding coefficient of each individual to assess the level of genetic diversity within each line.

### Functional annotation of variants

Annotation of the freebayes-called variants was performed using Variant Effect Predictor [28]. Variant effect prediction for protein-altering variants was performed using SIFT [29] and PROVEAN [30]. The following variant classes were considered as potentially causing loss of function: splice acceptor, splice donor, inframe indels, frameshift, stop loss, stop gain, and start lost variants. Moreover, only variants that were annotated in genes and which were (mostly) 1:1 orthologous in Ensembl (release 86) were retained to minimize the effect of off-site mapping of sequence reads, as this leads to miscalls, which can be particularly problematic for large gene families (e.g. olfactory receptors). In addition, compensation of function by (recent) paralogous genes will likely ameliorate the effects of damaging mutations in these genes. Also, since gene models might be incorrect, variants that did not have a combined RNA-seq expression coverage of at least 200 in the Ensembl (release 86) merged RNA-seq dataset were discarded. The number and load of deleterious variants for each line were inferred from the final set of deleterious variants.

### Spectrum of allele frequencies for different classes of variants

We determined the distribution of allele frequencies for different classes of variants (synonymous, missense tolerated, missense deleterious, stop-gained) to test whether predicted deleterious mutations have generally lower allele frequencies. We generated a histogram with 20 bins (with steps of 0.05 allele frequency) starting from a very low (0–0.05) to very high allele frequency (0.95–1) for the different classes of variants using the PyVCF and SciPy software packages.

### Candidate embryonic lethal variants in protein coding genes

To identify putative embryonic lethal (EL) variants, we selected all LoF and deleterious missense variants, for which no individuals that were homozygous for the alternate allele were observed. For every EL candidate we examined whether the gene is known to cause early lethality in mice obtained from the MGI database release 6.10 (i.e. phenotypes from null-mutant mice) [31]. We manually examined all predicted EL variants in JBrowse [32] to exclude false positives that derived from sequencing and mapping errors. Significant differences in hatchability between carrier by carrier versus carrier by non-carrier phenotypes were assessed using a two-sample t-test, assuming equal variances.

### Relative position of indels and stop-gained variants in the protein

We divided proteins from Ensembl release 86 in 10 bins (from N- to C-terminal end) and we determined the relative position of the indel and stop-gained variants by dividing the position of the affected amino acid by the total protein length.

### Fixed and line-specific “evolutionary-intolerant” variants

We considered all alleles with a frequency higher than 0.9 (within each line) as fixed or nearly fixed variation. To identify regions under selection, we used an approach similar to that described by Elferink et al. [33], but we applied a window size of 20 kb with a minimum number of 20 variants in each window. We selected a threshold of zHp ≤ − 2.7 representing the extreme lower end of the zHp distribution (see Additional file 2: Figure S1). Windows below this threshold were assumed to be enriched for regions of selective sweeps. We selected line-specific high-frequency variants (i.e. absent in the other two populations) with an allele frequency higher than 0.7.

### Gene-set enrichment analysis

We tested whether certain gene families are enriched for deleterious mutations. Therefore, gene-set enrichment analysis was performed using the DAVID functional annotation and classification tools [34]. Enrichment clusters (as produced by DAVID) with a score higher or equal to 1.3 were considered to be enriched [34].

### Deleterious alleles in regions of low recombination

The recombination rate is the genetic length in centimorgans divided by the physical genomic distance in mega base pairs and was calculated for bins of approximately 750 kb on macrochromosomes 1 to 5 using the linkage map of Elferink et al. [35]. Microchromosomes were excluded because of their extreme high recombination rates [36]. The ratio of predicted deleterious to predicted tolerated mutations (prediction by SIFT) was calculated within each bin by dividing the number of deleterious missense mutations by the sum of the synonymous and tolerated missense mutations over all three breeding lines. Pearson correlation was used to infer the relationship between the ratio of predicted deleterious to predicted tolerated mutations and the recombination rate.

## Results

### Screening for haplotypes that exhibit missing or deficient homozygosity

In layer breeding programs, genetic improvement is primarily achieved on elite purebred lines. These purebred lines are then crossed to produce parent stock production animals that are again crossed to produce the final laying hen production animals, which benefit from the full exploitation of heterosis [37]. To successfully screen these purebred lines for missing homozygosity, we assumed that not all deleterious variation has been purged, and that some low-frequency deleterious variation remains in the population. Since we examined carrier by carrier (C × C) matings, 25% of the offspring were expected to be homozygous for the carrier haplotype. In total, we examined six lines for missing homozygosity, one purebred white layer dam line (WA), one purebred white layer sire line (W1), two crossbred lines (CB: W1-WA, W1-WD) and two brown layer lines (B1, B2). In total, information was available for 22,323 (post-filtering) animals genotyped on the Illumina 60 K chicken SNP BeadChip (52,232 SNPs), which provided the statistical power required to detect even very rare haplotypes (see Additional file 1: Table S1). We performed phasing of all data to determine the haplotypes and used an overlapping sliding-window approach to identify haplotypes with a significant deficit in homozygotes.

We identified 9, 13, 7, and 50 haplotypes that exhibited a statistical deficit in homozygosity (DH) in the WA, W1, CB, and B1-B2 lines, respectively (Table 1) and (see Additional file 3: Table S1, S2, S3, and S4). The length of these haplotypes ranged from 0.25 to 1 Mb and the frequency of putative deleterious haplotypes ranged from 0.5 to 18.3%. The percentage of heterozygous progeny from C × C matings for these haplotypes was generally higher than 50%, which supports the deviation from HWE due to missing homozygous offspring (Table 1). The frequency of these haplotypes was generally low (< 5%) but two haplotypes that showed a deficit in homozygosity had relatively high frequencies (> 10%) in the crossbred line (on *Gallus gallus* chromosome (GGA)1: 180.25–180.75 Mb and GGA5: 5.5–6.0 Mb).

**Table 1:** Statistics for missing and depleted homozygous SNP haplotypes in four lines of layer chickens

Lines	WA	W1	CB	B1-B2
Samples	4409	7197	3983	6737
Trios	2291	3619	3539	3118
Number of haplotypes	9	13	7	50
Number of loci	9	13	7	45
Average haplotype length	24.22	33.3	22.29	23.20
Average number of haplotypes per window	17.11	15.08	12.43	15.40
Average haplotype frequency	2.6%	3.1%	8.3%	1.5%
Average homozygous expected	6.06	8.13	30.71	8.08
Average carrier matings with genotyped offspring	3.11	4.23	53.71	3.12
Average carrier matings in pedigree	9.00	12.38	54.71	6.62
Average carrier progeny	24.22	32.54	119.71	32.32
Percentage heterozygote carrier progeny	60.1%	51.3%	70.5%	46.0%
Average number of genes in window	20.9	20.0	9.14	6.30

Averages for all parameters are provided for each line. The number of loci represents the unique number of genomic windows containing significant haplotypes

We examined the sequence of the carriers for haplotypes showing a deficit in homozygosity (from the WA and W1 lines) for protein altering variants that were shared by the carriers for each putative deleterious haplotype but for which no homozygous individuals were observed. We identified two candidate mutations (see Additional file 1: Table S2) that segregated in the purebred (WA and W1) and crossbred lines. These two haplotypes, which were initially identified in the crossbreds (GGA2: 56.0–56.5, GGA3: 94.125–94.875 Mb), contain protein altering mutations in the *ADNP2* (C198S) and *SOX11* (A261G) genes. Both these genes are considered to be essential for normal development and associated with early lethality in mice (inferred from null-mutants, [38, 39]). Only the alanine to glycine mutation in the *SOX11* genes was predicted to be mildly deleterious by SIFT and PROVEAN (see Additional file 1: Table S2).

### A catalogue of genomic variation in three white-layer lines

We also explored the use of WGS data for direct inference of deleterious variation using sequence data from three commercial white layer lines, one sire line (W1), and two dam lines (WA and WD). We sequenced 250 animals from these lines (WA: 71, WD: 78, and W1: 101), for a total volume of 3.502 Tbp (tera base pairs) from 35.94 billion paired-end 100 bp reads. Mapping was performed with BWA-MEM (version 0.7.15, [24]) to the *Gallus gallus* build 5 reference genome [18] with an average mappability and coverage of 99.76%, and 11.4 (range: 8.3X to 22.9X), respectively (Pipeline overview [see Additional file 2: Figure S2]). We performed population-based variant calling using Freebayes [27] to identify 10,260,277 (post-filtering) variants in the three lines (see Additional file 1: Table S3). From the total 10,260,277 (post-filtering) identified variants, 9,469,408 (98.5% biallelic) were SNPs and 790,869 were indels. The average SNP density was 11.0 per kb (see Additional file 1: Table S3). We identified 2,143,367 novel variants (20.89%) that were not annotated in dbSNP (build 147), of which the majority was breeding line specific (WA, WD, or W1) (see Additional file 1: Table S4). An average call rate of 0.95 and an average transition/transversion (TS/TV) ratio of 2.53 were found for the entire variant set (see Additional file 2: Figure S3 and Additional file 1: Table S5), which are congruent with previous findings in other avian species [40, 41]. Sample origin was validated using principal component analysis (PCA) (see Additional file 2: Figure S4).

**Fig. 1:**
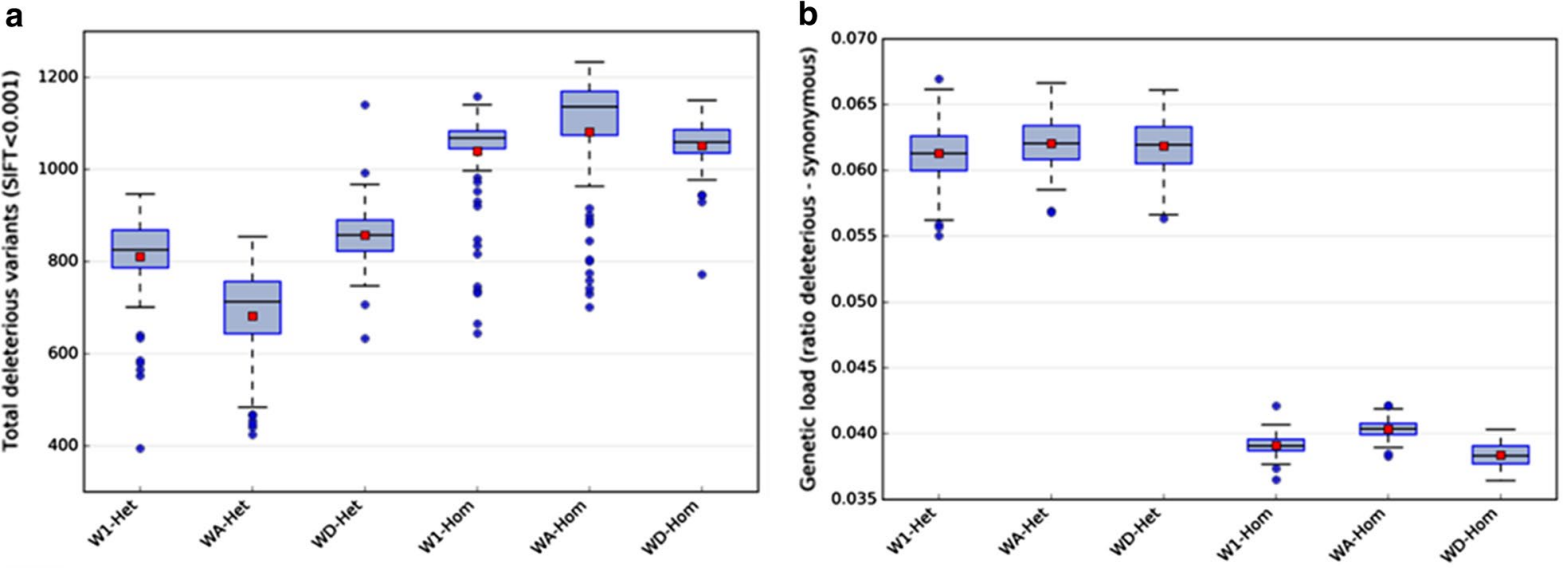
**a** Distribution of the number of heterozygous (-Het) and homozygous (-Hom) individuals for putative deleterious variants. **b** Mutation load, calculated as the ratio of deleterious to synonymous variants for heterozygous and homozygous individuals for putative deleterious variants

We assessed the level of genetic diversity by calculating the F statistic within the three lines (WA, WD, and W1) and observed that it was lower in the WA line than in the other two lines (see Additional file 2: Figure S5). Accordingly, we found a smaller number of line-specific SNPs in the WA line compared to the other two lines (see Additional file 1: Table S4). Moreover, we observed that WA animals carried on average fewer deleterious variants than the other two lines. However, the mutation load, calculated as the ratio of deleterious (SIFT < 0.01) to synonymous variants, was higher in the WA line than in the WD and W1 lines, which was in line with the lower genetic diversity within this line (Fig. 1).

Variant effect prediction assigned a range of functional classes to the identified variants (see Additional file 1: Table S6). Of the 120,149 coding (35,963 protein-altering) variants that we identified, the large majority were synonymous and non-synonymous mutations. Furthermore, 2.04% (2437) of the variants were classified as potentially introducing a loss-of-function (frameshift, inframe deletion, inframe insertion, splice acceptor, splice donor, start lost, stop gained, and stop lost variants). Of the 33,492 missense mutations, 5546 and 3053 were predicted to be deleterious by the SIFT and PROVEAN software, respectively, of which 1847 were predicted by both methods (see Additional file 2: Figure S6). A final set of 4219 putative deleterious variants, distributed across nine classes of deleterious variants, was obtained after filtering (see “Methods”) and (see Additional file 1: Table S7).

### Evidence for purifying selection on deleterious mutations

**Fig. 2:**
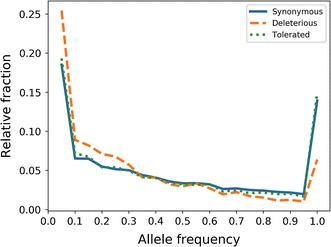
Allele frequency distribution for different functional classes of putative deleterious variants. Deleterious variants (deleterious missense and stop-gained) show distinct allele frequency spectra compared to variants considered to be neutral (synonymous, missense tolerated). Missense variants are classified by SIFT (deleterious: SIFT score ≤ 0.05, tolerated: SIFT score > 0.05)

We found that the spectrum of allele frequencies of deleterious variants differed from that of neutral variants, and was skewed towards a higher proportion of low-frequency alleles (Fig. 2) and (see Additional file 2: Figure S7). Their relative low frequency supports the hypothesis that the predicted deleterious variants are subject to purifying selection.

#### Relative position of indels and stop-gained variants in the protein

**Fig. 3:**
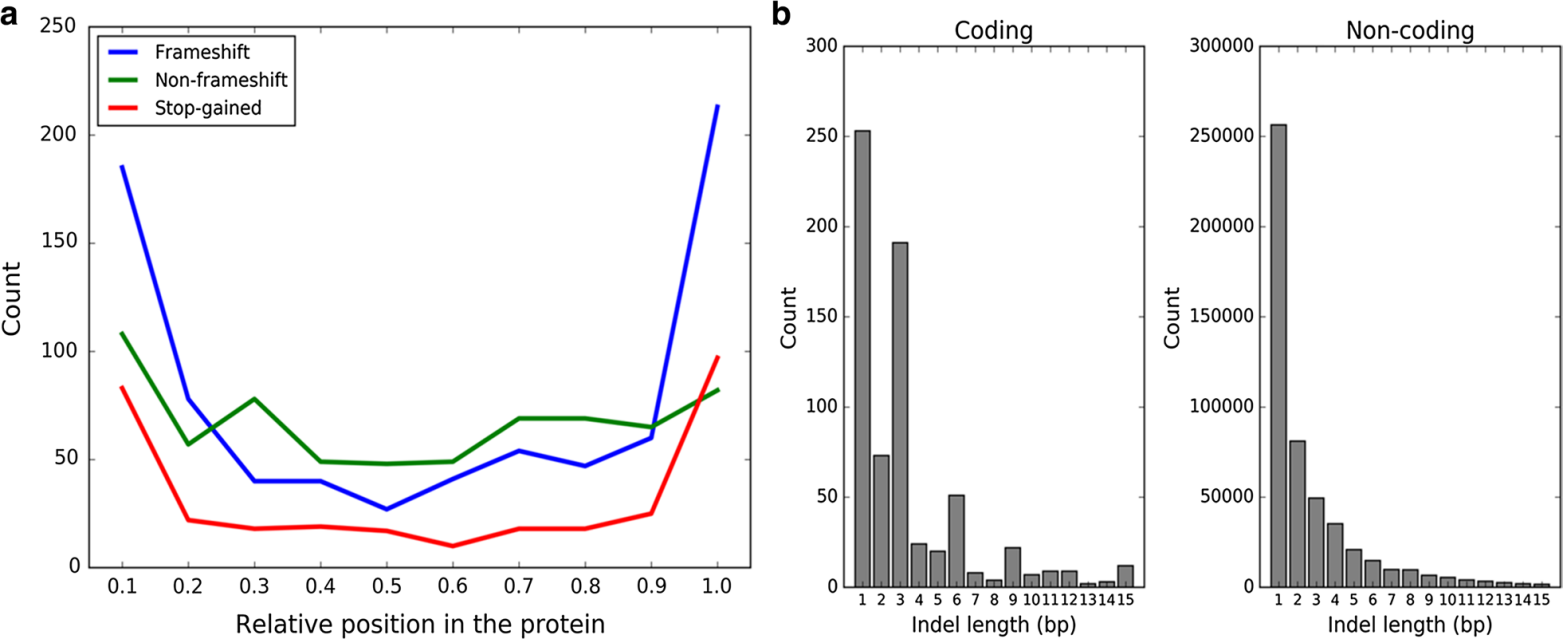
**a** Relative position of frameshift, non-frameshift indels, and stop-gained variants. Frameshift variants are enriched in N- and C-terminal parts of the protein. Frameshift variants at the N-terminal sites are potentially “rescued” by alternate start-codons. Frameshift variants at the C terminal end are likely not disruptive since a functional protein might still be translated. **b** Distribution of lengths of coding and non-coding indels. In-frame indels (i.e. indels with lengths of 3, 6, and 9 nucleotides) are enriched in coding regions

The impact of LoF variants on the protein is potentially determined by the position of the variant in the amino acid sequence. We found that frameshift and stop-gained variants were enriched at the N- and C-terminal ends of the protein, a pattern that was not present for inframe indels, which rather showed a more or less uniform distribution of location across the protein (Fig. 3a). Frameshift or stop-gained variants at the N-terminus could be “rescued” by alternate start-codons, while variants at the C terminus are less likely to be disruptive because they may still result in a more-or-less functional protein. Moreover, deleterious missense mutations occurred more often at the N- and C-terminal ends of the protein, while synonymous mutations occurred less frequently at those positions (see Additional file 2: Figures S8 and S9). Overall, coding indels were enriched for in-frame indels (e.g. 3, 6, 9 bp), because these are more likely to be evolutionary-tolerated (and therefore not purged from the population), which usually does not apply to frameshift indels (Fig. 3b).

#### Less effective purging in regions of low recombination

**Fig. 4:**
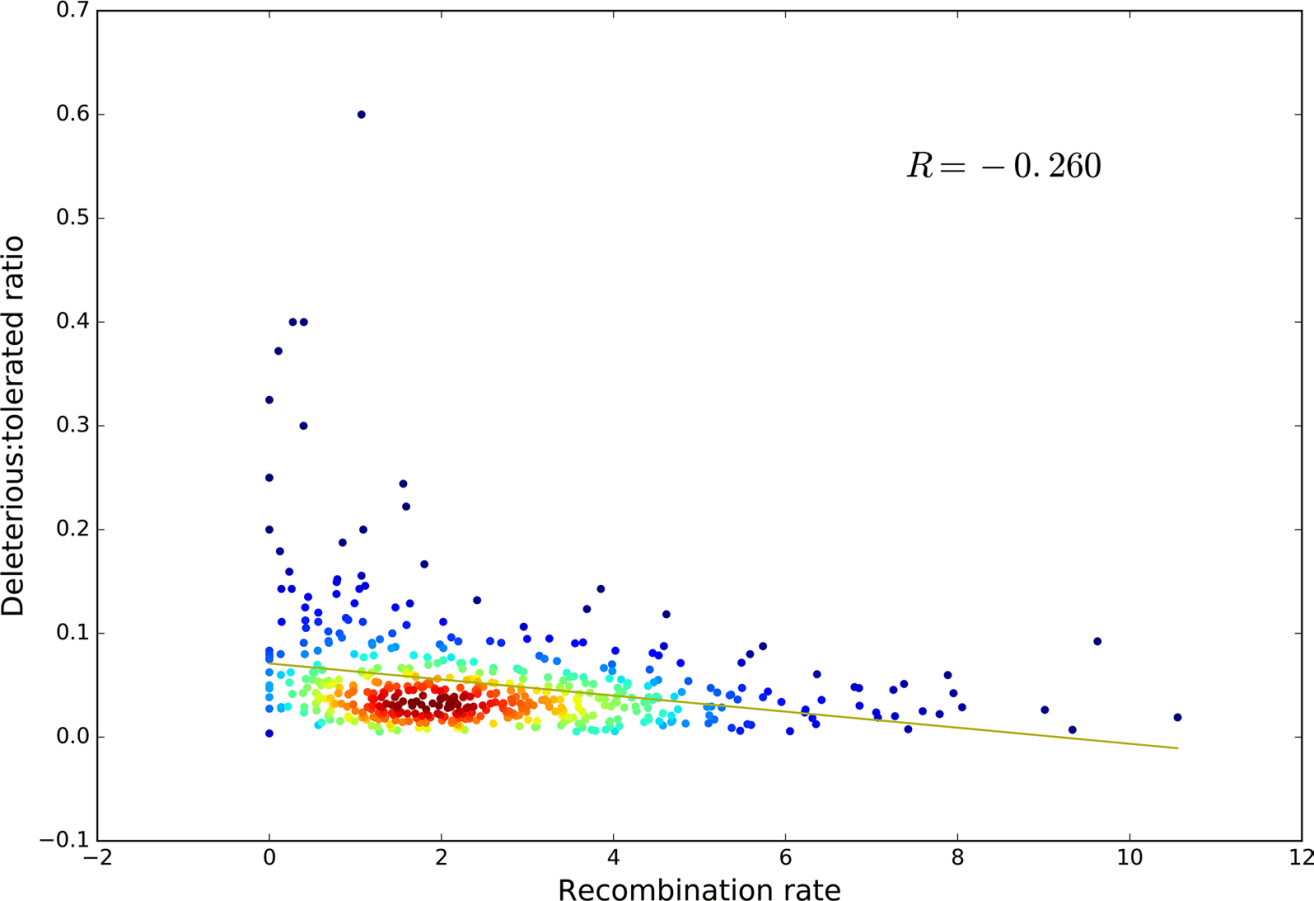
Pearson correlation between recombination rate and the ratio of putative deleterious to tolerated alleles for regions that harbour such alleles. Results indicate that regions of low recombination are generally enriched for deleterious variants (R = − 0.26, P = 2.89e−09)

Next, we examined whether the ratio of deleterious to tolerated mutations was affected by the recombination rate. A significant negative correlation (r = − 0.26, *p *= 2.89×10e^−9^) was found between the recombination rate and the ratio of deleterious to tolerated alleles, providing evidence of more effective purging in regions with high recombination rates (Fig. 4). Enrichment of deleterious over tolerated variants was especially evident in regions of very low recombination (recombination rate less than 2%, [see Additional file 2: Figure S10]).

### Candidate EL variants in protein coding genes

To identify variants that likely result in early lethality during development (EL), we selected all putative LoF and deleterious missense variants that met the following two criteria: (1) no homozygous individuals for the allele were observed; and (2) the affected gene caused early lethality in null-mutant mice [31]. Based on these criteria, we identified 11 frameshift, five inframe indels (predicted as deleterious by PROVEAN), six stop-gained, five splice acceptor, eight splice donor, and 121 deleterious missense variants (see Additional file 4: Table S1). The majority of these 152 candidate EL variants (86.6%) were specific to one line and contained frameshift mutations in the *APAF1* and *NHLRC2* genes, which are both associated with embryonic lethality and malformations in cattle [42, 43]. Of the five in-frame indels, two exhibited relatively high carrier frequencies (> 5%) in the WD line and affected the genes *CHTF18* and *FLT4*. We also identified 13 candidate splice donor and acceptor variants that could potentially lead to mis-splicing, resulting in an incomplete or incorrect protein. Two splice variants exhibited relatively high allele frequencies (> 5%) and affected the *POLR1B* and *HP1BP3* genes. Moreover, one high-frequency (22.3%) stop-gained variant affected the C-terminal end of the SCRIB protein and, thus, might not be disruptive as an almost complete functional protein should be translated (see Additional file 4: Table S1).

### Missense variants

The large majority (~ 84%) of the 122 candidate EL missense variants were specific to a line (WA: 19, WD: 46, and W1: 37). Twenty-five variants were predicted to be highly deleterious (PROVEAN score < − 5, Table 2, and [see Additional file 2: Figure S11]). One specific missense variant in the *OFD1* gene, which causes a tyrosine to cysteine substitution (Y19G), is a strong candidate for embryonic lethality in homozygous carriers, in spite of its relative high frequency (8.9%). The tyrosine at position 19 of OFD1 is highly conserved among vertebrates and, thus, this missense mutation is predicted to be highly deleterious (PROVEAN: − 7.42, SIFT: 0.0). From the 18 carrier animals (15 sires and 3 dams), we identified three C × C matings in the breeding data that showed a significant (*p *= 0.0165) increase in the percentage of embryos that died during development (see Additional file 1: Table S8).

**Table 2:** Missense variants predicted to be highly deleterious (PROVEAN score < − 5.0) and their phenotypic consequences in null mutant mice based on the MGI database

Chr.	Position	Ref.	Alt.	Heterozygotes	Line	Symbol	AA position	AA change	SIFT score	Provean score	MGI phenotype
1	49464407	C	T	7	W1	*NAGA*	271	R/C	0	− 6.42	Homeostasis
1	51524116	A	G	3	WA	*TMPRSS6*	688	E/G	0	− 5.771	Reproductive, growth/size/body, endocrine/exocrine, liver/biliary, immune, homeostasis, mortality/aging, integument, hematopoietic, digestive/alimentary
1	118357796	A	C	7	W1	*PRDX4*	81	F/V	0.02	− 5.65	Reproductive, cellular, endocrine/exocrine
1	122963712	T	C	18	W1	*OFD1*	19	Y/C	0	− 7.42	Embryo, nervous, system, skeleton, craniofacial, limbs/digits/tail, renal/urinary, respiratory, cellular, mortality/aging, cardiovascular, growth/size/body, digestive/alimentary
2	66538263	T	A	9	WA	*BPHL*	70	D/V	0	− 8.815	Hearing/vestibular/ear, homeostasis
3	108278046	C	T	3	WD	*PKHD1*	3397	R/W	0.03	− 5.066	Respiratory, growth/size/body, endocrine/exocrine, liver/biliary, renal/urinary, cellular, mortality/aging, cardiovascular, nervous system, hematopoietic, digestive/alimentary
4	51931175	G	T	4	W1	*CENPC*	729	P/Q	0	− 7.783	Embryo, mortality/aging, growth/size/body, cellular
4	62191743	T	C	3	WD	*FAT1*	796	Y/C	0.01	− 7.534	Nervous system, craniofacial, renal/urinary, vision/eye, mortality/aging, pigmentation, growth/size/body, homeostasis
4	70191405	G	A	15	WD	*TBC1D1*	182	R/C	0	− 6.635	Growth/size/body, adipose, cellular, no abnormal phenotype observed, muscle, homeostasis
5	58052925	G	A	7	W1	*NIN*	1206	R/C	0.02	− 6.045	Hearing/vestibular/ear, nervous system, behaviour, cardiovascular
5	58235842	C	T	8	W1	*NID2*	944	G/S	0	− 5.151	Immune, skeleton
9	17236801	C	A	3	WA	*CCDC39*	857	P/H	0	− 5.599	Respiratory, skeleton, craniofacial, liver/biliary, immune, renal/urinary, homeostasis, cellular, mortality/aging, digestive/alimentary, growth/size/body, hematopoietic, cardiovascular
9	17571682	C	A	3	WD	*MFN1*	439	G/W	0	− 7.551	Embryo, mortality/aging, growth/size/body, cellular
19	4443604	A	T	11	WD	*UNC45B*	225	I/N	0	− 5.4	Mortality/aging
19	6266804	C	T	9	WD	*CPD*	994	P/L	0.01	− 6.728	Respiratory, behaviour, reproductive, craniofacial, endocrine/exocrine, liver/biliary, immune, digestive/alimentary, homeostasis, cellular, vision/eye, integument, nervous system, skeleton, growth/size/body, hematopoietic, cardiovascular
24	4359027	G	A	3	WA	*KMT2A*	1941	P/L	0	− 9.253	Embryo, liver/biliary, muscle, cellular, reproductive, immune, craniofacial, limbs/digits/tail, hearing/vestibular/ear, renal/urinary, neoplasm, homeostasis, behaviour, cardiovascular, mortality/aging, integument, nervous system, growth/size/body, hematopoietic, skeleton
27	3474503	C	A	4	WA	*MPP3*	206	S/Y	0	− 5.649	Nervous system, vision/eye, cellular

### Fixed evolutionary-intolerant variants include potential selection candidates

We identified 473 predicted deleterious alleles that were fixed (247) or nearly fixed (allele frequency > 90%) in the three white layer lines (WA, WD, and W1) (see Additional file 5: Table S1). Gene-set enrichment analysis showed that the corresponding genes are involved in energy metabolism (e.g. ATP-binding, calmodium-binding) and muscle and motor activity (see Additional file 5: Table S2). Several of these variants were strongly selected in domesticated chicken. For example, variant (G558R) in the *TSHR* gene was completely fixed in all three lines and this mutant allele is associated with the absence of strict regulation of seasonal reproduction found in natural populations [16]. A deleterious inframe deletion (108delE) was also found in the *P2RY2* gene, which is an ATP receptor. In addition, 12 fixed deleterious variants were identified in seven myosin-related genes (*MYH7B*, *MYCBPAP*, *MYO1G*, *MYH9*, *MYLK3*, *MYO9B*, and *MYLK2*) that are involved in skeletal muscle development [44]. Other gene families that contained fixed deleterious variants were the protein-tyrosine-phosphatases (*PTPN7*, *PTPRJ*, *TNS3*, *PTPRE*, *PTPRF*, and *DUSP28*), the centrosome proteins (*CEP97*, *CEP162*, *CEP89*, and *CEP164*), which are potentially involved in essential developmental processes, based on evidence of early lethality in knockout model organisms (notably *CEP97* and *CEP164,* [31]), and collagen-like genes (e.g. *C1QTNF8*, *C1QTNF6*, *EMILIN2*). Forty variants in 37 genes were predicted to have a severe impact on the protein produced by these genes (PROVEAN score $\le$ − 5), including a variant in the *TSHR* gene (see Additional file 5: Table S3).

#### Selection candidates

To distinguish between true selection candidates and effects of genetic drift, we examined the populations for regions under selection. Genome-wide Z-scores of heterozygosity (zHp) were calculated per 20-kb windows. We considered bins with a zHp less than − 2.7 as potential regions of selective sweeps in the genome (representing the extreme end of the distribution) (see Additional file 2: Figure S1) and found 27 fixed evolutionary intolerant variants in these regions (see Additional file 2: Figure S12 and Additional file 5: Table S4), which overlap with the *TSHR* (see Additional file 2: Figure S13) and *FOXI1* genes, previously described as being under domestication selection [16, 17].

We focussed on predicted evolutionary-intolerant variants in smaller regions of selective sweeps to identify possible functional variation that has been under selection. We identified a splice donor variant in the *CPE* gene (see Additional file 2: Figure S14), which is involved in the energy metabolism of cells and insulin processing. In addition, we identified a strong selection signal in two bins that overlapped with a missense variant in the *CCDC93* gene (T389 M) (see Additional file 2: Figure S15). This gene is involved in protein transport, but, although various quantitative trait loci (QTL) related to egg production and egg quality overlap with this gene [45], its exact function remains unknown. A splice acceptor variant in the *PSMC6* gene, a start lost variant in the *GLCCI1* gene, and an inframe insertion in the *RUNXT1* gene were identified as potential additional functional target mutations (see Additional file 2: Figures S16, S17 and S18). *PSMC6* and *GLCCI1* are both involved in energy metabolism, and overlap respectively with an egg shell thickness QTL and a QTL for haugh unit (a measure of egg protein quality based on the height of its egg white) and growth [45]. The *RUNX1T1* gene is a transcription factor involved in the generation of precursor metabolites (substances from which energy is derived). All these variants are likely functional, and while they are identified as being damaging in a natural or wild context, they may have been favourably selected for because they positively affect desired traits in egg-laying hens.

### Line-specific high-frequency deleterious variation

#### WA breeding line

We found 26 high-frequency (allele frequency > 0.7) deleterious missense variants, one frameshift and three splice variants specific to the WA breeding line. Interestingly, the *ASPM* gene contains three deleterious missense variants (see Additional file 6: Table S1). This gene encodes a mitotic spindle protein and is expressed in proliferating tissues and is associated with a range of phenotypes, including decreased body weight, microcephaly, and reduced fertility in both sexes. Two variants were predicted to have a severe impact on CIB1 (R112C) and PCSK6 (R87 W) proteins (PROVEAN score < − 5), which are both involved in mammalian fertility. CIB1 is related to abnormal spermatogenesis, decreased testis weight and male infertility, while PCSK6 showed a role in female fertility (ovary cysts, increased ovary tumour incidence) [31].

#### WD breeding line

We annotated 77 high-frequency deleterious variants specific to the WD breeding line (see Additional file 6: Table S2), which included 59 deleterious missense variants, one inframe deletion (*ENSGALG00000030853*), 14 splice acceptor/donor variants, one start-loss variant (*PCBD2*), and two stop-gained variants (*BRIC5* and *NCOR1*). Interestingly, the *FYCO1* gene, which is associated with cataract phenotypes in mammals [31], harbours two highly deleterious missense variants. Moreover, six missense variants are predicted to be highly deleterious by PROVEAN (*PIGX*, *CARMIL2*, *LPAR6*, *ENSGALG00000015226*, *LIMK2*, *RIC3*). Three of these genes were demonstrated to have severe effects in null-mutant mice (*CARMIL2*, *LPAR6*, and *LIMK2*) [31].

#### W1 breeding line

We identified 35 high-frequency variants specific to the W1 breeding line (see Additional file 6: Table S3), which included 31 deleterious missense variants, three splice-donor variants, and one stop-gained variant (*NOLC1*). Three missense variants in three different genes (*TAAR1*: Y290 N, *VWA1*: P251S, *MCM10:* P39L) were predicted to be highly deleterious. *TAAR1,* a trace amine associated receptor gene, and *VWA1* are both associated with various behavioural traits, including increased hyperactivity (*TAAR1*) and abnormal motor coordination/balance (*VWA1*). Null-mutants for the *MCM10* gene are embryonic lethal in mammals, resulting in abnormal growth prior to termination of development [31]. Interestingly, the *CSPG4* gene harbours three deleterious missense variants in the W1 line, which are associated with abnormal muscle cell physiology and increased body weight [46].

## Discussion

Combining a systematic genomic survey for missing homozygosity and whole-genome sequence (WGS) data opens new opportunities to directly infer functional variants. We have presented a first full genomic catalogue of variants that provides a perspective on the deleterious and functional variation in fairly closed, and relatively inbred, purebred layer lines. We not only confirmed previous “domestic” or selective variants but also assessed the impact of deleterious variation in these lines. Taken together, this genomic framework can be used to further improve and understand the genomic elements that are selected or purged in current breeding programs. Finally, a better understanding of the variants with functional implications will provide a useful resource for further selection programs to help distinguish true deleterious variants from those with positive functional implications.

Domesticated populations are expected to be under artificial selection against inbreeding depression. Indeed, in this paper, we show that putatively highly deleterious (i.e. lethal) variants are rare in the commercial chicken populations studied here, in spite of the small effective size of these populations. However, we found several examples of putative lethal variants with allele frequencies up to 10% (e.g. OFD1 and Y19C) and showed that, although under strong selection, the purging of these variants is not always very effective, even in modern poultry breeding programs. Artificial selection in these populations may be ‘strong’, but is based on an index of a large number of phenotypic traits. Balancing selection may also be acting on these populations (e.g. heterozygote advantage), which causes deleterious variants to remain in the population.

In order to capture deleterious variants using haplotypes of SNPs that exhibit missing homozygosity, the low-frequency haplotype has to be in complete LD with the causal variant. However, most deleterious variants (EL) reside on common haplotypes that cannot be detected with medium-density SNP chip data. However, absence of specific homozygous allele states can now be inferred directly because animals can be routinely genotyped for these variants, such that they can be added to the currently used genomic selection framework. A similar study in cattle showed that 15% of the LoF and 6% of the tested missense variants are likely true EL [15]. Although predicting EL variation from sequence can be sensitive to induce false positives, we tried to reduce the number of false positives by manually examining the predicted EL variants. Moreover, the distinct allele frequency spectrum for our predicted deleterious mutations compared to neutral mutations confirms that they are subject to purifying selection.

One limitation of our study is that we focused on coding variation, however, a large proportion of the non-coding genome is also subject to purifying selection because of their biological function [47]. As a result, we may have missed a large proportion of potential deleterious or functional variants. In addition, livestock genomes still lack proper annotation of many functional elements but currently there are many efforts to improve this aspect [48].

We found no evidence of a higher load of deleterious variants in our studied chicken lines compared to other livestock species [15, 49]. However, although the impact of individual variants on the population may be limited, a recent study showed that negative selection involves synergistic epistasis, which means that the combined effect of mutations is greater than the sum of the individual effects. This supports the hypothesis that the overall effect of the deleterious mutations on population fitness might be substantial [50]. As a consequence, the number of deleterious variants found in the chicken populations studied here might represent a universal level for ‘healthy populations’, i.e. lower levels deleterious mutations are not attained because selection against low-frequency alleles is ineffective, but higher levels of deleterious mutations could occur, which then rapidly leads to disproportionately large inbreeding depression effects. This study also demonstrates the value of domesticated populations to provide insight in the genomic architecture of inbreeding depression and can be useful for future studies on inbreeding in both wild and domesticated populations.

The observed spectrum of allele frequencies for predicted deleterious and tolerated variants corroborates the hypothesis that the predicted deleterious variants (especially deleterious missense and stop-gained variants) have been under purifying selection. Conversely, the predicted tolerated missense variants followed the same distribution of allele frequencies as synonymous variants (usually considered to be neutral), which indicates that the large majority of these predicted missense variants are indeed evolutionary tolerated. Within coding regions, we also found an enrichment of indels that are multiples of three nucleotides, which was not the case for non-coding indels. Indels that alter the frame of translation in coding regions can be highly disruptive, for instance by introducing a premature stop codon and, therefore, such indels are often under purifying selection. Conversely, indels that are multiples of three nucleotides will result in losses or gains of one or multiple amino acid residues, which have a higher likelihood of being tolerated. We also observed an enrichment of frameshift and stop-gained variants at the N- and C terminal ends of the protein, which suggests that, in general, these types of variants have a stronger impact on the function of the protein when they are located in the middle part of the protein compared to the distal parts of the protein. Namely, if they are located at the N-terminal part of the protein, a functional protein product might still be generated by an alternate start codon that can “rescue” a large part of the protein (N-terminal part), as described previously [51]. In contrast, a frameshift or stop-gained variant at the C-terminal end may be tolerated since an almost complete protein is often generated. Together these genomic signatures of purifying selection support our predictions on deleterious alleles within the populations.

Evidence that the frequency of recombination in a genomic region is negatively correlated with the ratio of deleterious to tolerated mutations suggests more effective purging in regions with higher recombination rate, potentially because deleterious variants that hitchhike along with selected variants are more easily physically disconnected from variants that are under selection in regions with high recombination rates. Similar results have been reported in other species, although always with weaker correlations [2, 3, 52]. We shed light on the role of recombination (i.e. more effective selection in regions of high recombination) in genomic purging within the avian clade, which is known for its highly diverse recombination rates between chromosomes, with notably extremely high recombination rates on microchromosomes [53].

In addition to predicted deleterious variants with low frequencies, several high-frequency predicted deleterious variants were identified that likely have high functional relevance. We focussed on predicted evolutionary-intolerant, but high-frequency, variants in selective sweep regions. This study confirmed several predicted deleterious variants that were previously identified as being positively selected in domesticated chicken populations, e.g. variants in the *TSHR* and *FOXI1L* genes [16, 17]. However, we find several novel predicted deleterious variants in strong selective sweep regions (e.g. variants in the *CCDC93*, *PSMC6* and *GLCCI1* genes), that should be further investigated for phenotypic effects. In spite of a paucity of functional annotation, there is evidence that the majority of these genes have a role in cellular energy metabolism and likely cause increased metabolic activity [16, 33].

The use of genomic selection has increased the rate of genetic improvement in breeding populations substantially over the past years [6]. However, genomic selection remains a “black-box” approach and the genomic architecture that underlies selection remains unknown. Without additional prior information on the functional effects of low-frequency variants, effective selection for or against desired or unwanted variation remains challenging. Leveraging low-frequency functional variants for selection requires functional annotation, which can then be translated into statistical priors in enhanced genomic selection programs [54–56]. This study contributes to this by the identification of specific variants that can be incorporated in breeding programs to enhance genetic improvement.

## Conclusions

In this study, we applied several methods to infer deleterious variation in three commercial white-layer lines. We confirmed that missing homozygosity can result from lethal variants that reside on low-frequency SNP haplotypes. We were able to capture even very low-frequency deleterious variation, including 152 likely EL variants, by exploiting WGS data of dozens of sequenced individuals within single populations. Results provided clear evidence for purifying selection, based on a distinct spectrum of allele frequencies of deleterious variants compared to that of variants that have a higher likelihood of being neutral. In spite of their low-frequency nature, the identified putative deleterious alleles generally occurred more often in regions with low recombination, which suggests that purging of these alleles is less effective in such regions. Also, frameshift and stop-gained variants were more frequent at the protein N- and C-termini, which confirms that these are likely evolutionary-tolerated, which also applies to in-frame indels. In addition, multiple predicted evolutionary intolerant coding variants were discovered in selective sweep regions, which are likely under positive selection. A comprehensive genomic catalogue of putative deleterious variants was developed for white-egg layer breeding lines, which can enhance current genomic breeding practices to lower the frequency of undesirable variants in the population.

## Additional files

**Additional file 1: Table S1.** Statistics per breed. **Table S2.** Candidate mutations in regions of missing homozygosity. **Table S3.** Single nucleotide variants (SNV) and indels called. **Table S4.** Summary of identified variants for each line. **Table S5.** Call rate and TS/TV for all variants after filtering. **Table S6.** Functional annotation of variants (VEP). **Table S7.** Final list of deleterious variants identified in nine different classes. **Table S8.** Fertility phenotypes for three CXC matings that carry the OFD1 (Y19C) missense mutation.

**Additional file 2: Figure S1.** (A) zHp value distribution; (B) Genome-wide zHp values for the three white-layer lines. **Figure S2.** Pipeline overview for the detection of deleterious and functional variants using population WGS data. **Figure S3.** Chromosome distribution of transition/transversion (TS/TV) ratio across all sequenced animals. **Figure S4.** PCA analysis for the sequenced samples in WA, WD, and W1 lines. **Figure S5.** Population F-statistics. **Figure S6.** Pearson correlation of SIFT and PROVEAN scores on 13,065 missense mutations. **Figure S7.** Allele frequency distribution of different classes of variants**. Figure S8.** Relative position of tolerated and deleterious missense variants (SIFT). **Figure S9.** Relative position of synonymous, tolerated, and deleterious missense variants (SIFT). **Figure S10.** Distribution of the ratio of deleterious to tolerated alleles in two classes of recombination rates (<=2, >2). **Figure S11.** Missense variants predicted to be highly deleterious (PROVEAN <= -4.0) with no homozygotes observed. **Figure S12.** Fixed evolutionary-intolerant variants in selective sweep regions. **Figure S13.** Selective sweep region comprising the *TSHR* gene. **Figure S14.** Selective sweep region comprising the *CPE* gene. **Figure S15.** Selective sweep region comprising the *CCDC93* gene. **Figure S16.** Selective sweep region comprising the *PSMC6* gene. **Figure S17.** Selective sweep region comprising the *GLCC1* gene. **Figure S18.** Selective sweep region comprising the *RUNX1T1* gene.

**Additional file 3: Table S1.** Haplotypes exhibiting missing or depleted homozygosity in the WA line. **Table S2.** Haplotypes exhibiting missing or depleted homozygosity in CB lines. **Table S3.** Haplotypes exhibiting missing or depleted homozygosity in the W1 line. **Table S4.** Haplotypes exhibiting missing or depleted homozygosity in the B1-B2 line.

**Additional file 4: Table S1.** Candidate EL variants.

**Additional file 5: Table S1.** Evolutionary-intolerant fixed variants (AF > 90%). **Table S2.** DAVID gene-set enrichment analysis for evolutionary-intolerant fixed variation. **Table S3.** Evolutionary-intolerant variants predicted to have a severe impact on the protein (PROVEAN score <= -5). **Table S4.** Fixed evolutionary-intolerant variants in selective sweep regions.

**Additional file 6: Table S1.** High-frequency deleterious variation in the WA line. **Table S2.** High-frequency deleterious variation in the WD line. **Table S3.** High-frequency deleterious variation in the W1 line.
