# Supplementary material for: A survey of functional genomic variation in domesticated chickens
Source: Genet Sel Evol. 2018 Apr 16;50:17. doi: 10.1186/s12711-018-0390-1 (PMC5902831; doi:10.1186/s12711-018-0390-1)
Supplement: Supplementary file 1 — Additional file 1: Table S1. Statistics per breed. Table S2. Candidate mutations in regions of missing homozygosity. Table S3. Single nucleotide variants (SNV) and indels called. Table S4. Summary of identified variants for each line. Table S5. Call rate and TS/TV for all variants after filtering. Table S6. Functional annotation of variants (VEP). Table S7. Final list of deleterious variants identified in nine different classes. Table S8. Fertility phenotypes for three CXC matings that carry the OFD1 (Y19C) missense mutation. [file 12711_2018_390_MOESM1_ESM.pdf]

Additional file 1

Table S1: Statistics per breed.

Breed	# 60K Genotyped individuals	# Animal post-filtering	# Trios	Markers post filtering
W1	7,197	7,195	3,619	28,637
WA	4,409	4,408	2,291	21,412
Crossbreds	3,983	3,983	3,539	42,304
B1_B2	6,737	6,737	3,118	37,560
Total	22,326	22,323	12,567	-

Table S2: Candidate mutations in regions of missing homozygosity.

CHR	Start (Mb)	Stop (Mb)	Line	Candidate variant	Sift	Provean
**Chr2**	56.0	56.5	CB	56228028 C>G Intragenic (ADNP2) C>S	0.5	2.28
**Chr3**	94.125	94.875	CB	94938357C>G Intragenic (SOX11) A>G	0.2	-1.815

Table S3: Single nucleotide variants (SNV) and indels called.

Chromosome	SNV	Indel	Variants	Chromosome length	Var/Kb
Chr1	1,971,111	169,649	2,140,760	196,202,544	10.911
Chr2	1,486,364	132,306	1,618,670	149,560,735	10.823
Chr3	1,058,154	96,426	1,154,580	111,302,122	10.373
Chr4	922,021	86,916	1,008,937	91,282,656	11.053
Chr5	620,753	51,964	672,717	59,825,302	11.245
Chr6	448,712	34,075	482,787	35,467,016	13.612
Chr7	398,061	32,354	430,415	36,946,936	11.650
Chr8	300,013	23,751	323,764	29,963,013	10.805
Chr9	270,342	20,788	291,130	24,091,566	12.084
Chr10	212,953	16,484	229,437	20,435,342	11.227
Chr11	191,204	15,863	207,067	20,218,793	10.241
Chr12	218,662	16,807	235,469	19,948,154	11.804
Chr13	188,765	13,637	202,402	18,407,460	10.996
Chr14	169,624	12,743	182,367	15,595,052	11.694
Chr15	110,915	8,272	119,187	12,762,846	9.339
Chr16	6,599	286	6,885	652,338	10.554
Chr17	111,023	7,421	118,444	10,956,400	10.810
Chr18	114,860	7,758	122,618	11,053,727	11.093
Chr19	96,983	6,728	103,711	9,979,828	10.392
Chr20	142,351	9,975	152,326	14,109,371	10.796
Chr21	71,739	4,940	76,679	6,862,722	11.173
Chr22	24,781	1,807	26,588	4,729,743	5.621
Chr23	60,640	3,730	64,370	5,786,528	11.124
Chr24	71,099	4,360	75,459	6,280,547	12.015
Chr25	20,497	1,146	21,643	2,906,300	7.447
Chr26	58,179	3,560	61,739	5,313,770	11.619
Chr27	61,154	3,286	64,440	5,655,794	11.394
Chr28	49,981	3,116	53,097	4,974,273	10.674
Chr33	11,868	721	12,589	1,648,031	7.639
**Total**	**9,469,408**	**790,869**	**10,260,277**	**932,918,909**	**10.998**

Table S4: Line summary of identified variants. Novel variants were not present in dbSNP version 147.

	WA	WD	W1	WA_WD	WA_W1	WD_W1	WA_WD_W1	Overall
**Variants**	728,436	1,157,962	1,084,834	501,501	735,794	982,922	5,068,828	10,260,277
**Known VARs**	406,979	726,101	694,685	427,160	630,810	853,156	4,378,019	8,116,910
**Novel VARs**	321,457	431,861	390,149	74,341	104,984	129,766	690,809	2,143,367
**% Novel VARs**	44.13	37.29	35.96	14.82	14.27	13.20	13.63	20.89

Table S5: Call rate and TS/TV for all variants after filtering.

Chromosome	Call rate	TS	TV	TS/TV
Chr1	0.944692	1129177	450334	2.51
Chr2	0.944304	844945	334280	2.53
Chr3	0.945744	603364	232126	2.6
Chr4	0.945532	526798	203058	2.59
Chr5	0.950012	386771	144338	2.68
Chr6	0.956276	273390	106793	2.56
Chr7	0.955404	244603	90565	2.7
Chr8	0.952972	187062	66997	2.79
Chr9	0.956952	164844	59965	2.75
Chr10	0.955428	132331	46157	2.87
Chr11	0.951136	117524	42576	2.76
Chr12	0.955512	136799	47661	2.87
Chr13	0.95844	120664	42106	2.87
Chr14	0.95624	109004	37016	2.94
Chr15	0.957284	70656	23239	3.04
Chr16	0.815256	5015	2685	1.87
Chr17	0.960776	70915	22790	3.11
Chr18	0.94992	74016	26073	2.84
Chr19	0.958604	64273	20885	3.08
Chr20	0.958244	91152	30545	2.98
Chr21	0.956732	46518	15382	3.02
Chr22	0.94512	16774	5564	3.01
Chr23	0.950632	40665	12612	3.22
Chr24	0.961016	45112	14177	3.18
Chr25	0.891384	13412	4327	3.1
Chr26	0.946696	38697	11916	3.25
Chr27	0.894332	42426	18632	2.28
Chr28	0.924016	32957	10700	3.08
Chr33	0.9091	8077	2588	3.12
Overall:	0.948088	5637941	2126087	2.651792

Table S6: Functional annotation of variants (VEP)

Functional class	Total
3 prime UTR variant	65375
5 prime UTR variant	10357
Frameshift variant *	518
Inframe deletion *	185
Inframe insertion *	181
Splice acceptor variant *	549
Splice donor variant *	516
Start lost *	142
Stop gained *	275
Stop lost *	71
Mature miRNA variant	108
Upstream gene variant	647537
Downstream gene variant	485199
Missense variant	33492
Synonymous variant	84096
Non coding transcript exon variant	35226
Protein altering variant	34
Coding sequence variant	90
Splice region variant	17769
Stop retained variant	35
Intergenic variant	4156199
Intron variant	4722323
Sum (all variants)	10260277
Sum (LoF)	2437

* LoF: splice acceptor variant, inframe deletion, splice donor variants, inframe insertion, stop lost, stop gained, start lost, frameshift variant.

Table S7: Final list of deleterious variants identified in nine different classes.

Variant class / Breed	WA	WD	W1	WA_WD	WA_W1	WD_W1	WA_WD_W1	Sum
**Splice acceptor variant**	14	32	33	24	37	40	281	461
**Inframe deletion**	4	10	22	13	10	11	69	139
**Splice donor variant**	20	42	35	22	25	45	240	429
**Inframe insertion**	4	13	9	9	7	11	61	114
**Stop lost**	1	3	0	1	1	4	11	21
**Stop gained**	5	16	9	5	12	7	33	87
**Start lost**	4	5	2	1	2	3	26	43
**Frameshift variant**	9	19	16	12	13	10	107	186
**Deleterious missense**	208	420	311	174	259	297	1070	2739
**Sum**	269	560	437	261	366	428	1898	4219

Table S8: Fertility phenotypes for three CxC matings that carry the OFD1 (Y19C) missense mutation.

Eggs Set	Embryos Died	Eggs Fertile	Eggs Transferred	Total Hatched	%Fertility	%Hatch of Fertile	%Hatch of Set	%Hatch of Transferred	%Embryo died
12	2	12	10	8	100.00%	66.67%	66.67%	80.00%	16.67%
11	2	11	9	8	100.00%	72.73%	72.73%	88.89%	18.18%
13	1	12	11	9	92.31%	75.00%	69.23%	81.82%	7.69%
				**CxNC (N=402)**	**86.52**	**84.57%**	**73.93%**	**87.25%**	**2.83%**
				**W1 Overall**	**84.28%**	**83.29%**	**71.09%**	**86.51%**	**3.29%**
