# Supplementary material for: A survey of functional genomic variation in domesticated chickens
Source: Genet Sel Evol. 2018 Apr 16;50:17. doi: 10.1186/s12711-018-0390-1 (PMC5902831; doi:10.1186/s12711-018-0390-1)
Supplement: Supplementary file 2 — Additional file 2: Figure S1. (A) zHp value distribution; (B) Genome-wide zHp values for the three white-layer lines. Figure S2. Pipeline overview for the detection of deleterious and functional variants using population WGS data. Figure S3. Chromosome distribution of transition/transversion (TS/TV) ratio across all sequenced animals. Figure S4. PCA analysis for the sequenced samples in WA, WD, and W1 lines. Figure S5. Population F-statistics. Figure S6. Pearson correlation of SIFT and PROVEAN scores on 13,065 missense mutations. Figure S7. Allele frequency distribution of different classes of variants. Figure S8. Relative position of tolerated and deleterious missense variants (SIFT). Figure S9. Relative position of synonymous, tolerated, and deleterious missense variants (SIFT). Figure S10. Distribution of the ratio of deleterious to tolerated alleles in two classes of recombination rates (<=2, >2). Figure S11. Missense variants predicted to be highly deleterious (PROVEAN <= -4.0) with no homozygotes observed. Figure S12. Fixed evolutionary-intolerant variants in selective sweep regions. Figure S13. Selective sweep region comprising the TSHR gene. Figure S14. Selective sweep region comprising the CPE gene. Figure S15. Selective sweep region comprising the CCDC93 gene. Figure S16. Selective sweep region comprising the PSMC6 gene. Figure S17. Selective sweep region comprising the GLCC1 gene. Figure S18. Selective sweep region comprising the RUNX1T1 gene. [file 12711_2018_390_MOESM2_ESM.pdf]

Additional file 2

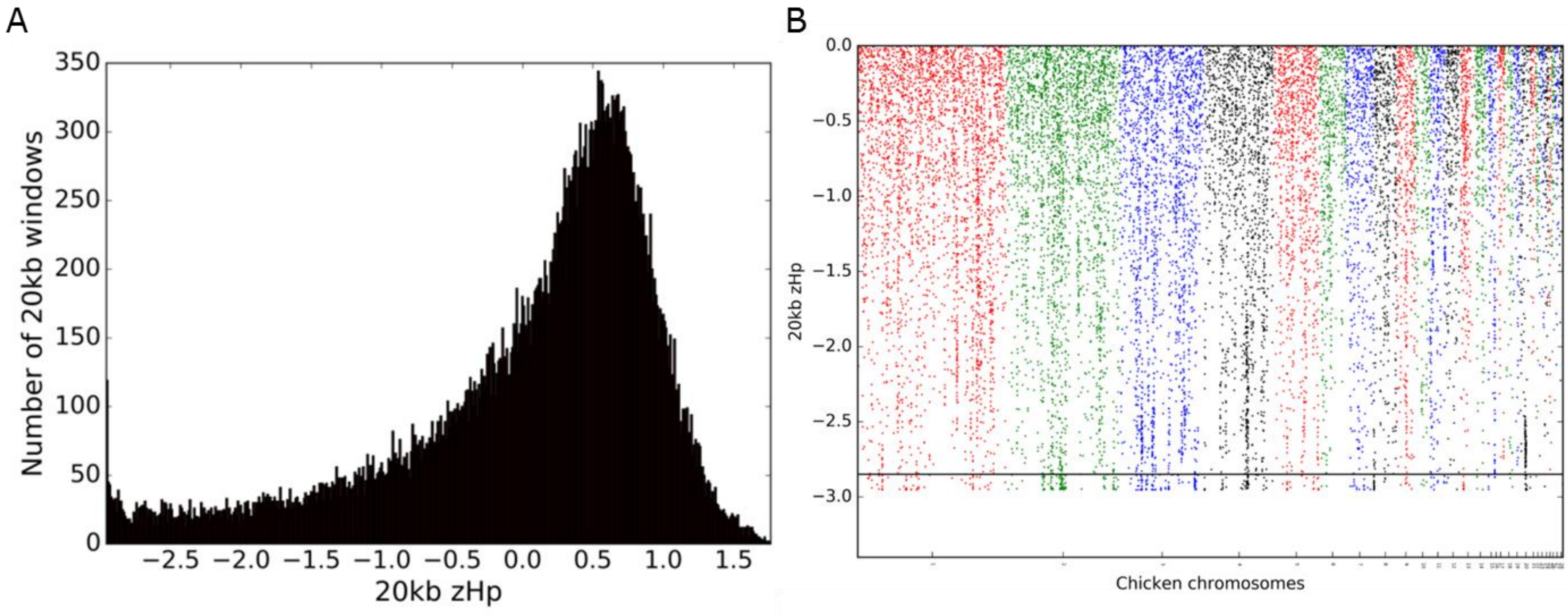

Figure S1: A) zHp value distribution. B). Genome wide zHp values for the three white layer lines. Bins with a zHp score <= -2.7 are examined for fixed deleterious variation.

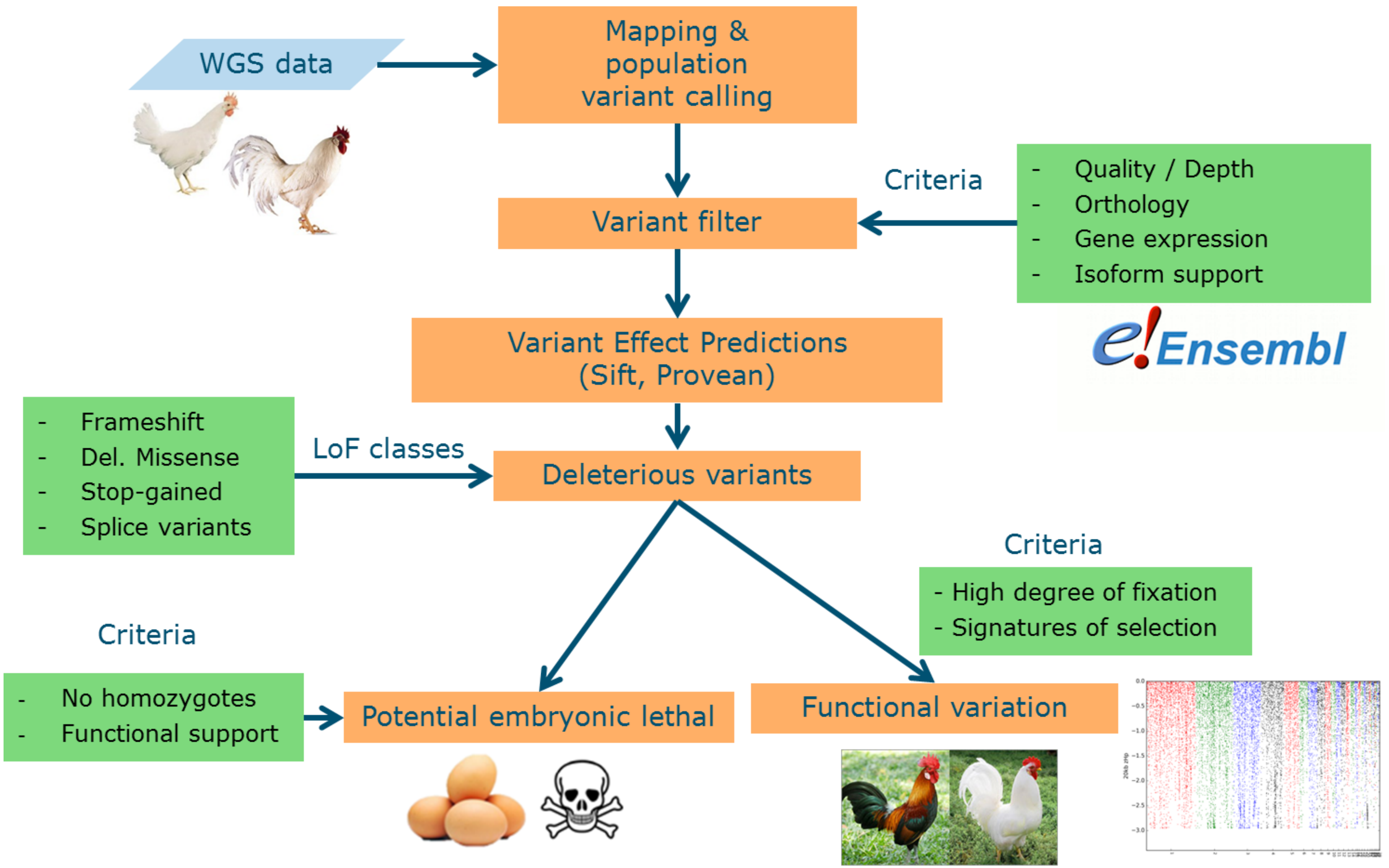

Figure S2: Pipeline overview to detect deleterious and functional variants using population WGS data.

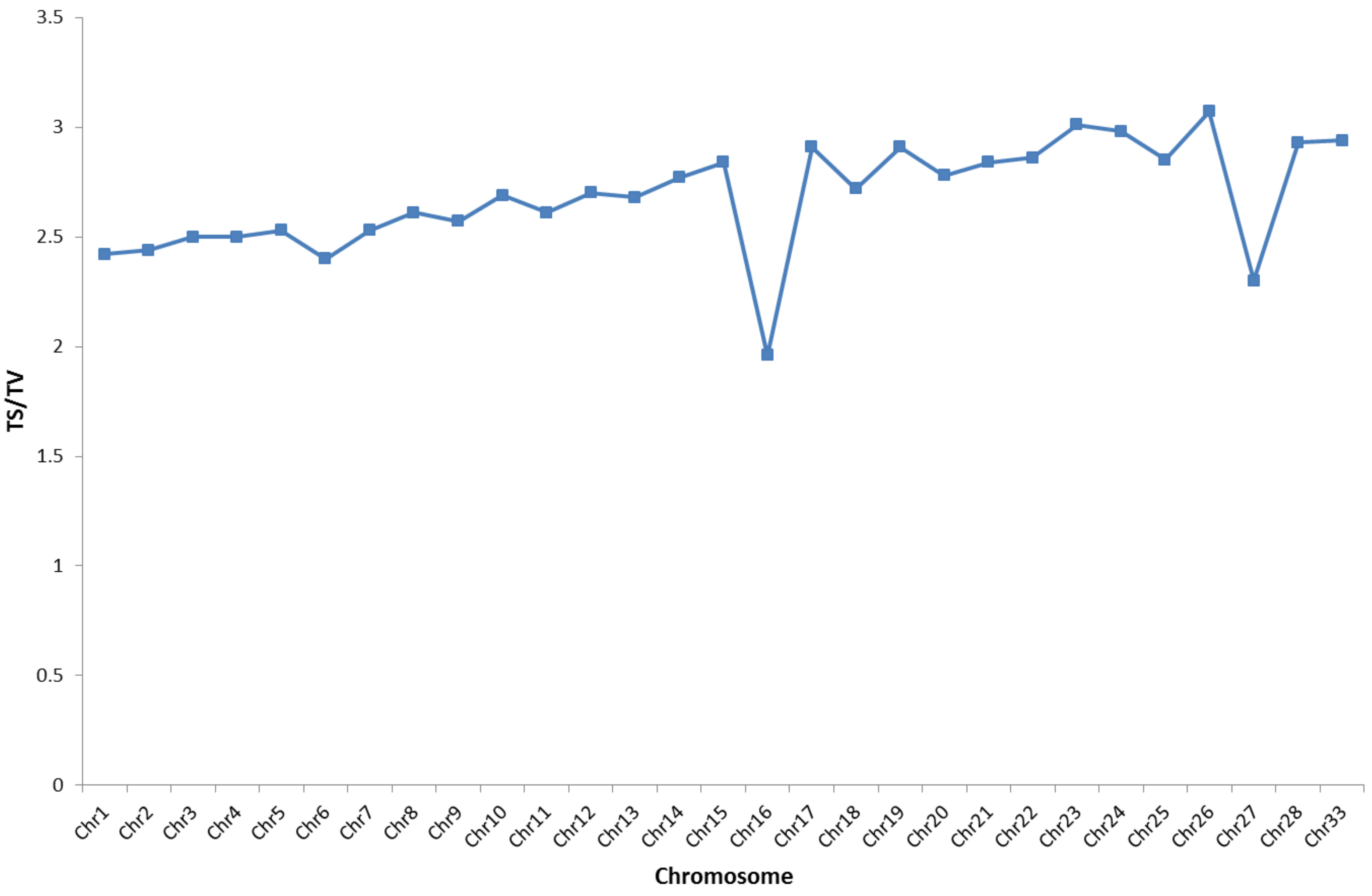

Figure S3: Chromosome distribution of transition/transversion (TS/TV) ratio over all sequenced animals.

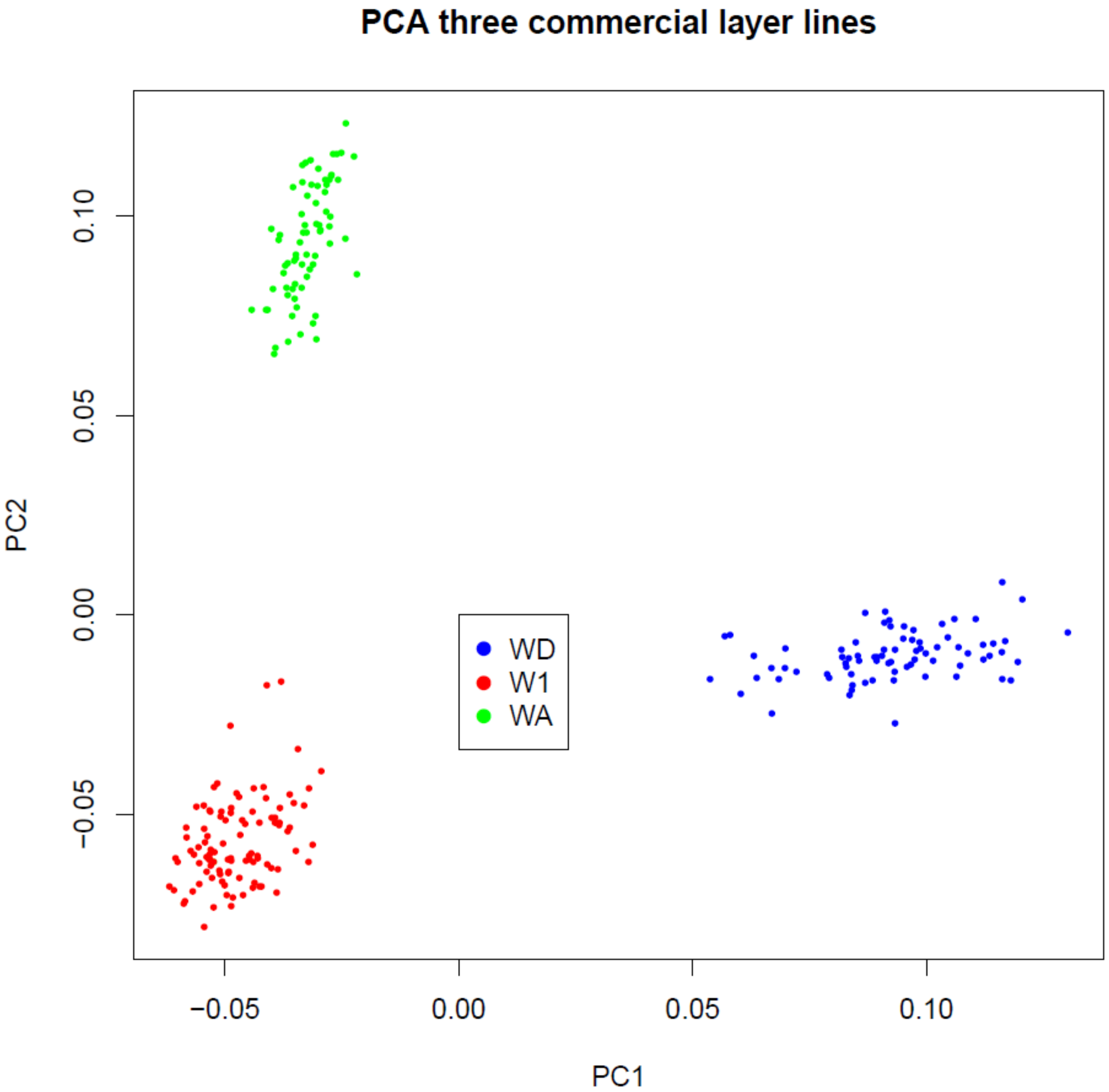

Figure S4: PCA analysis for the sequenced samples in WA, WD, and W1 breed.

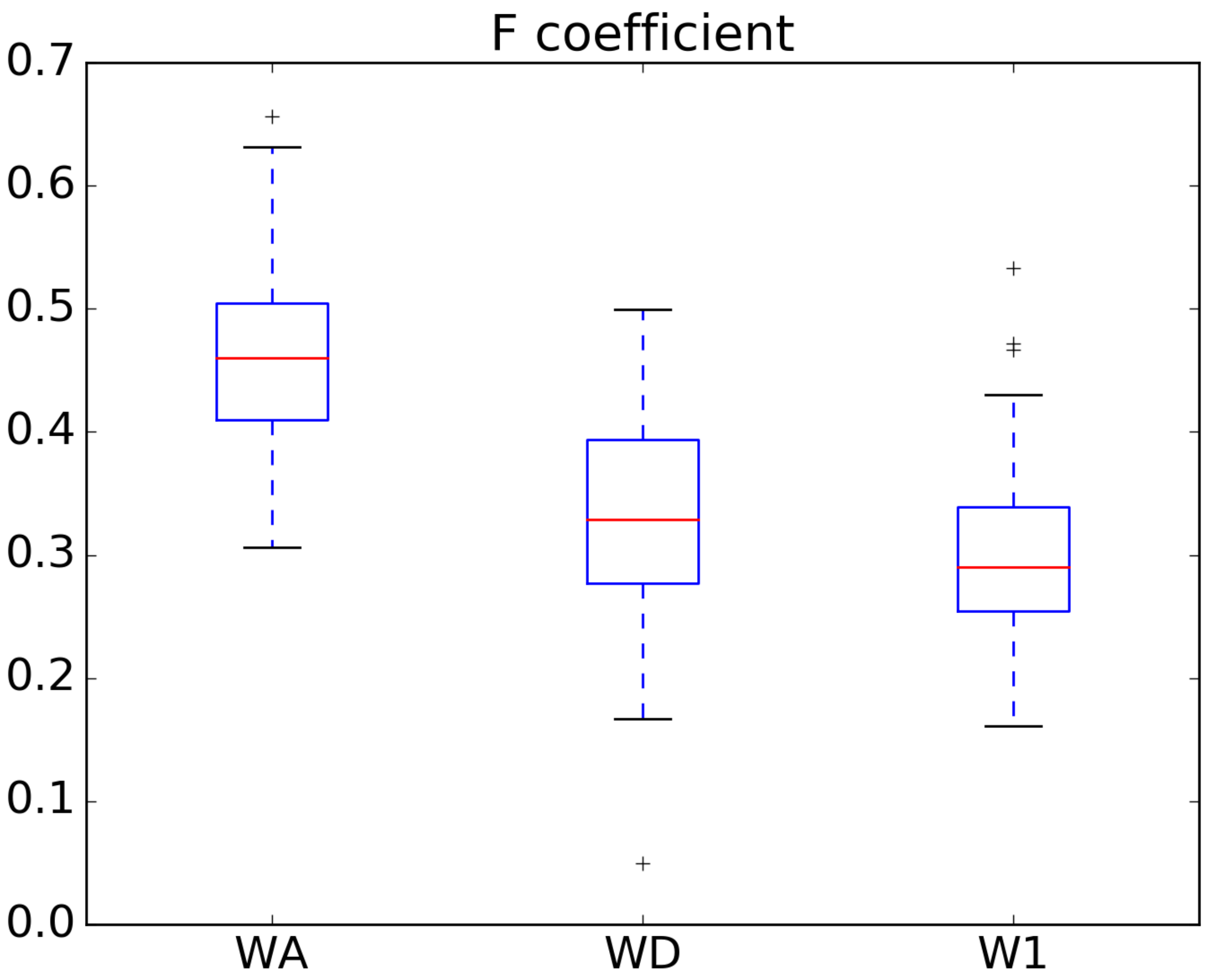

Figure S5: Population F-statistic. The inbreeding F-statistic (observed vs expected homozygous genotype counts) was calculated from the toal number of variants identified on chromosome 1.

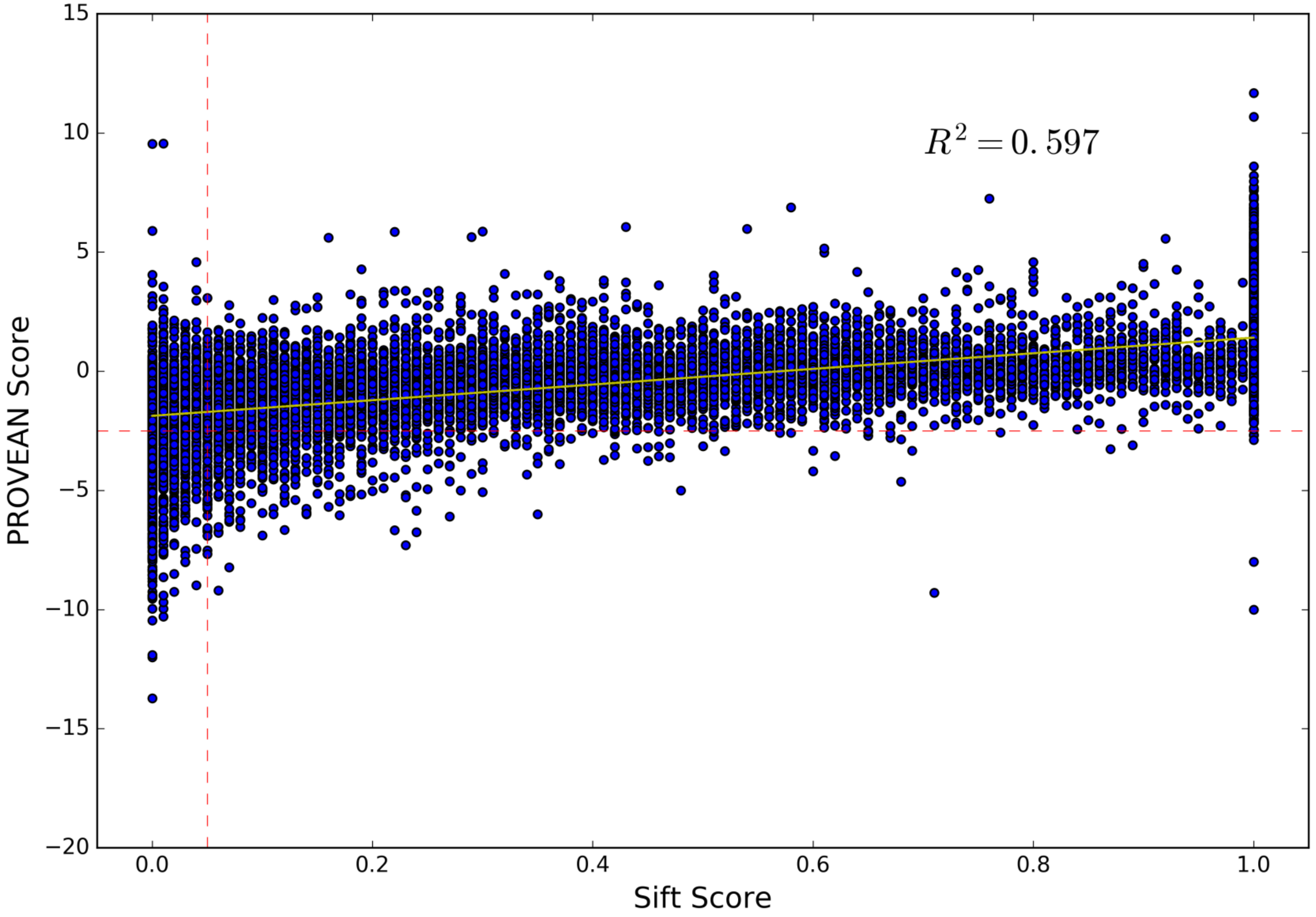

Figure S6: Pearson correlation of SIFT and PROVEAN scores on 13,065 missense mutations. A variant is considered to be deleterious if the score is below the given threshold (SIFT 0.05, PROVEAN -2.5).

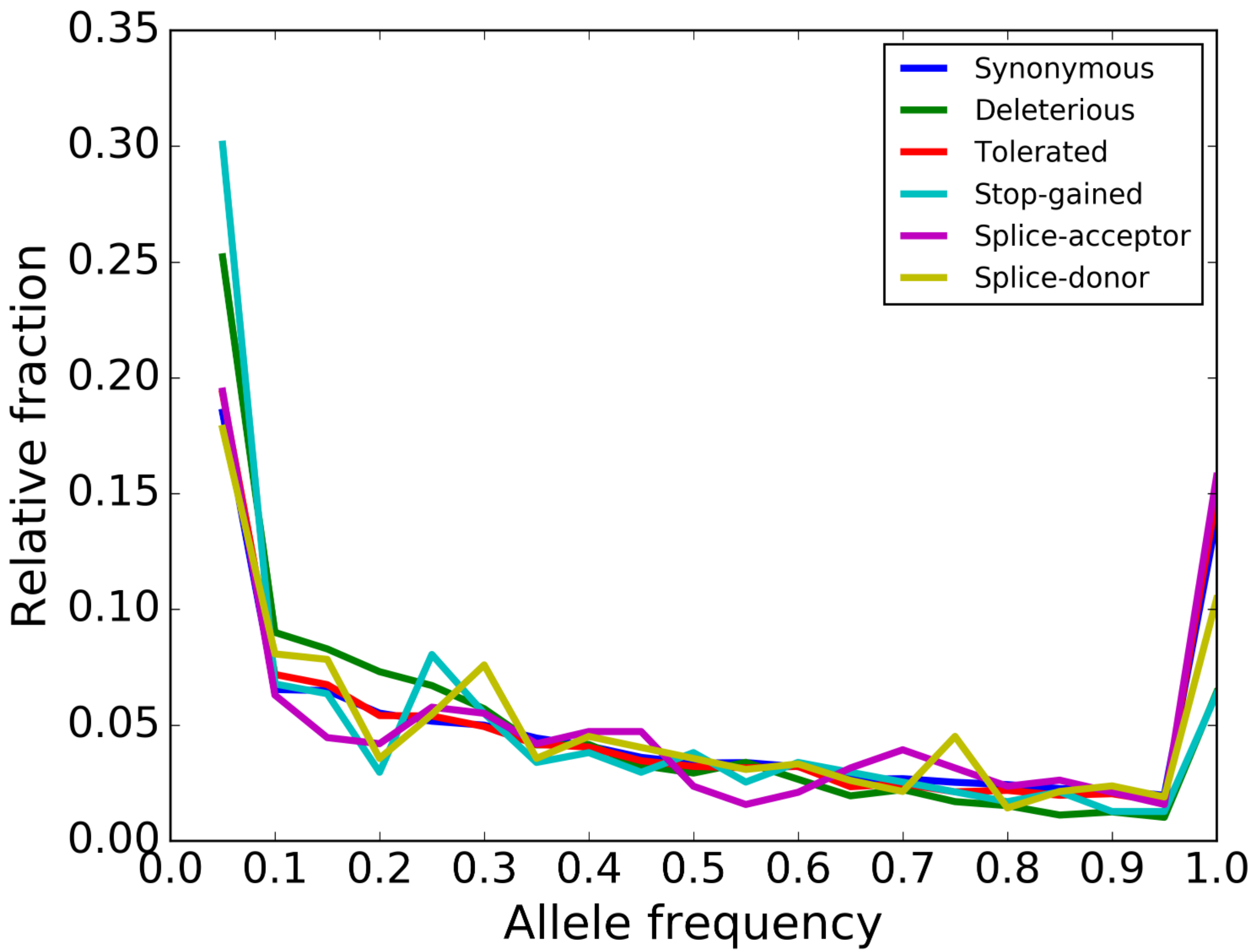

Figure S7: Allele frequency distribution of different classes of variants. Deleterious and stop-gained variants show a distinct allele frequency pattern showing that these variants were subject to purifying selection.

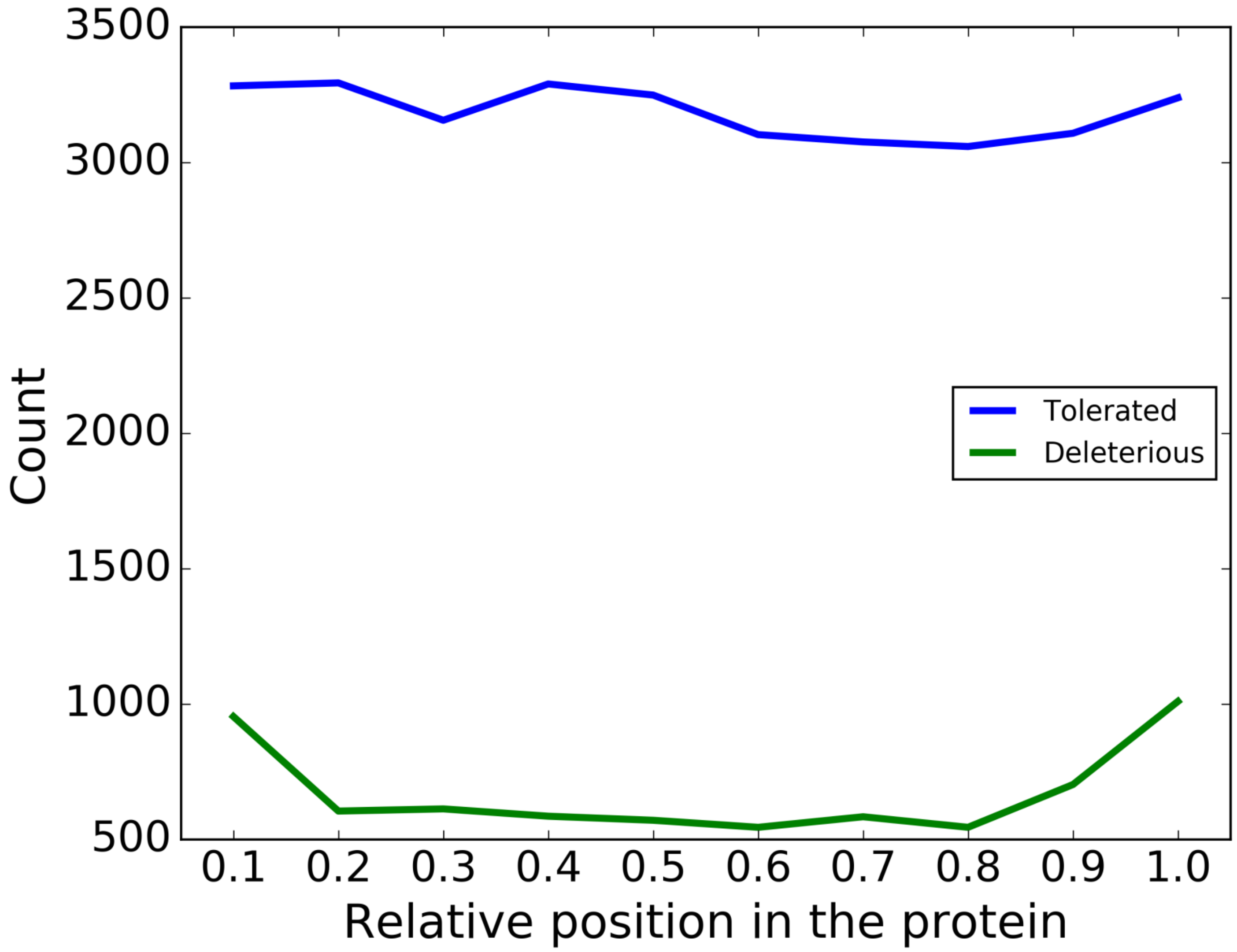

Figure S8: Relative position of tolerated and deleterious missense variants (SIFT). Deleterious variants are enriched in N- and C-terminal parts of the protein.

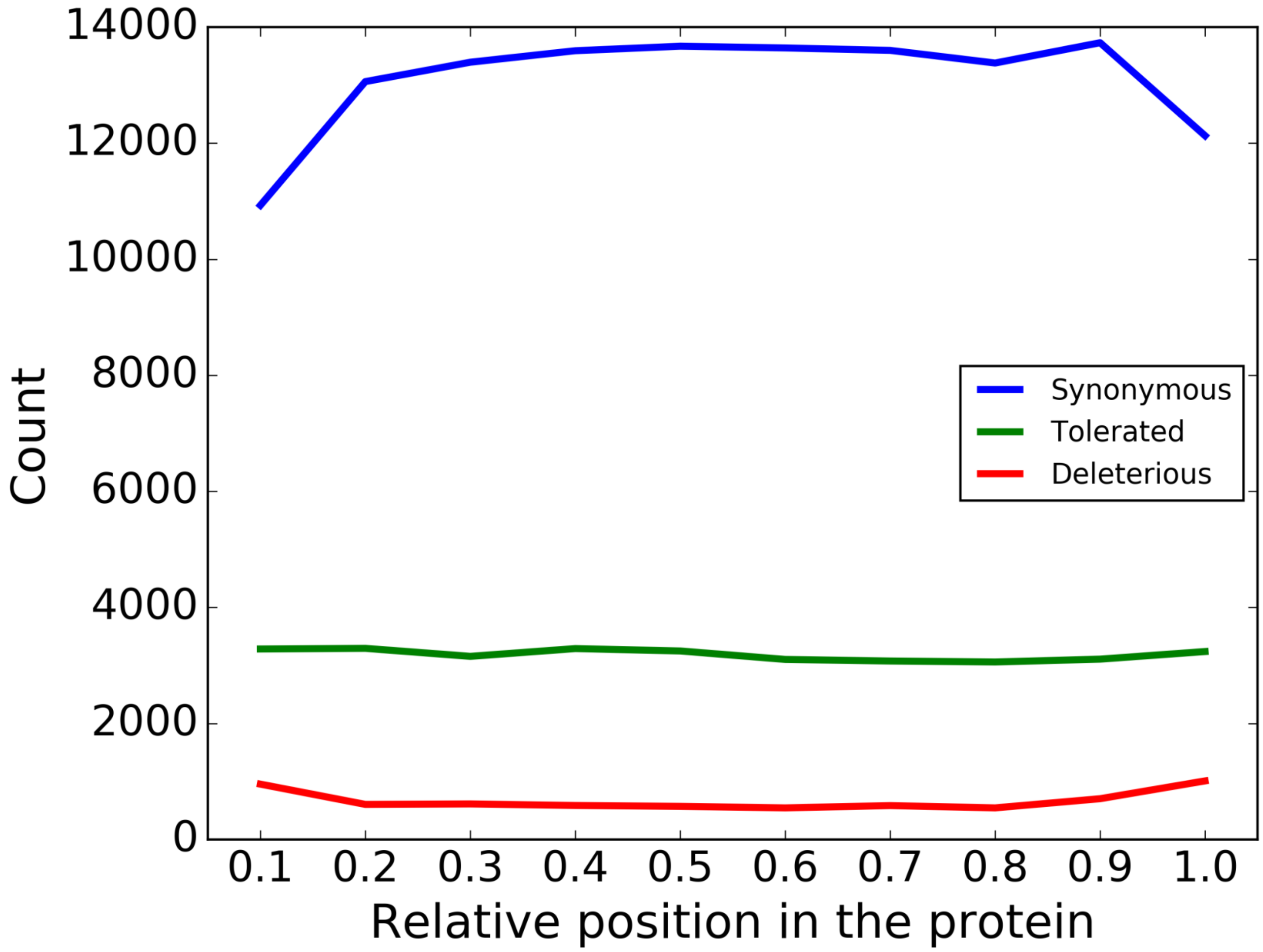

Figure S9: Relative position of synonymous, tolerated, and deleterious missense variants (SIFT). Synonymous variants occur less frequent in the N- and C-terminal part of the gene.

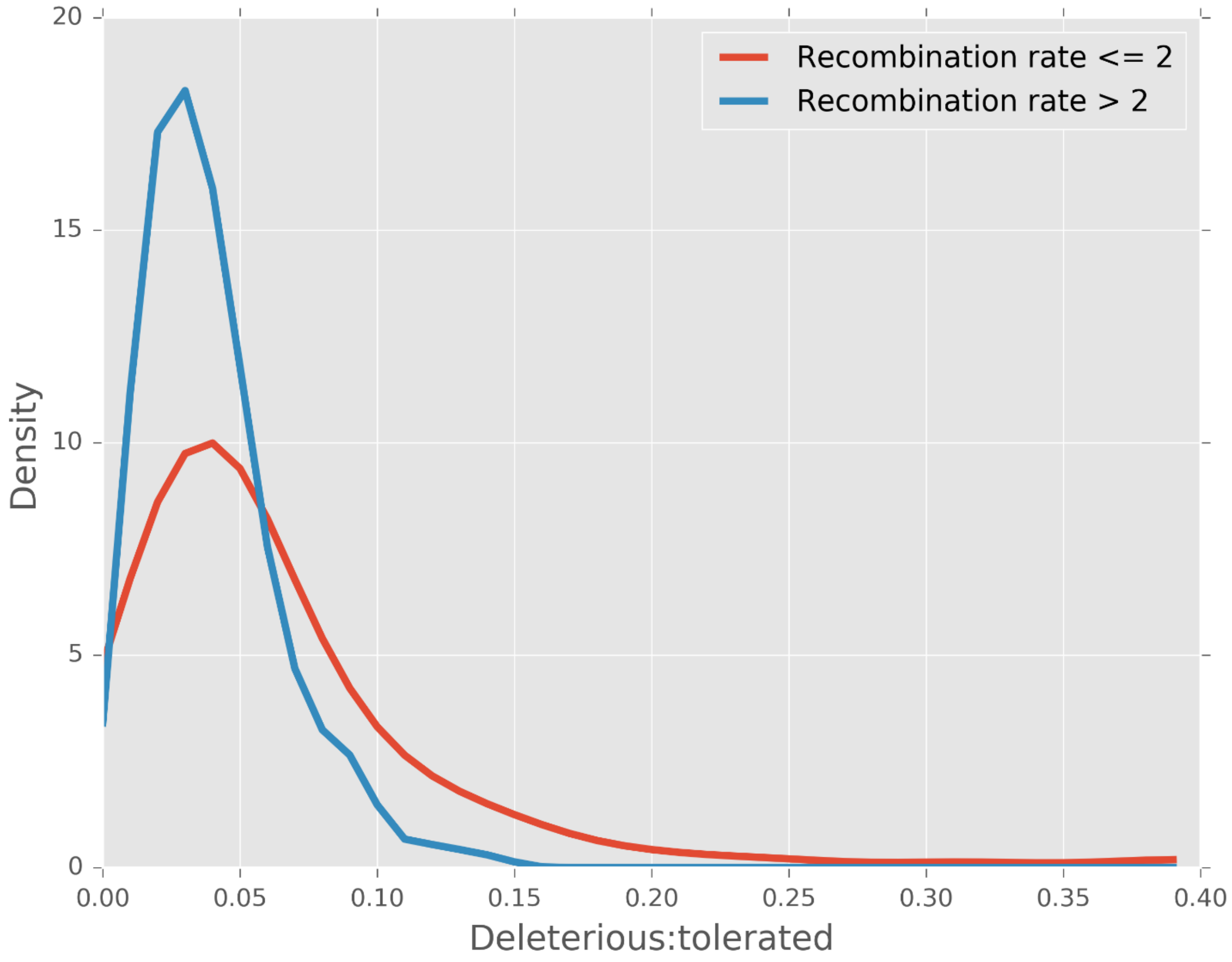

Figure S10: Distribution of the ratio deleterious to tolerated alleles in two classes of recombination rates (<=2, >2).

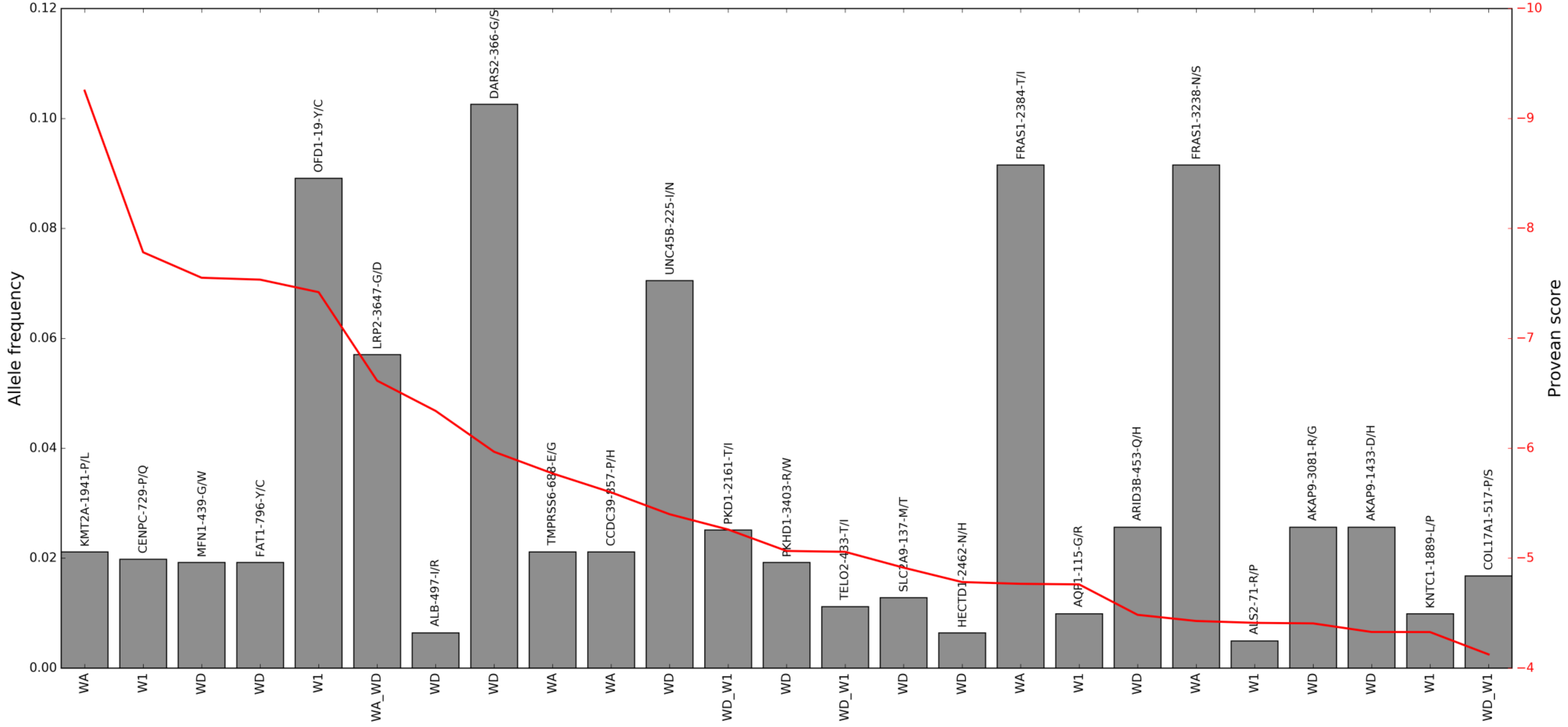

Figure S11: Missense variants predicted to be highly deleterious (PROVEAN <= -4.0) with no homozygotes observed. The bars indicate the allele frequency (left y-axis), the red line indicated the provean score (right y-axis). The breed in which a specific variant is present is indicated on the x-axis. All mutations are located in genes considered to be essential for normal development in mice (inferred from null mutants) and are potentially embryonic lethal in homozygous state (Blake et al. 2017).

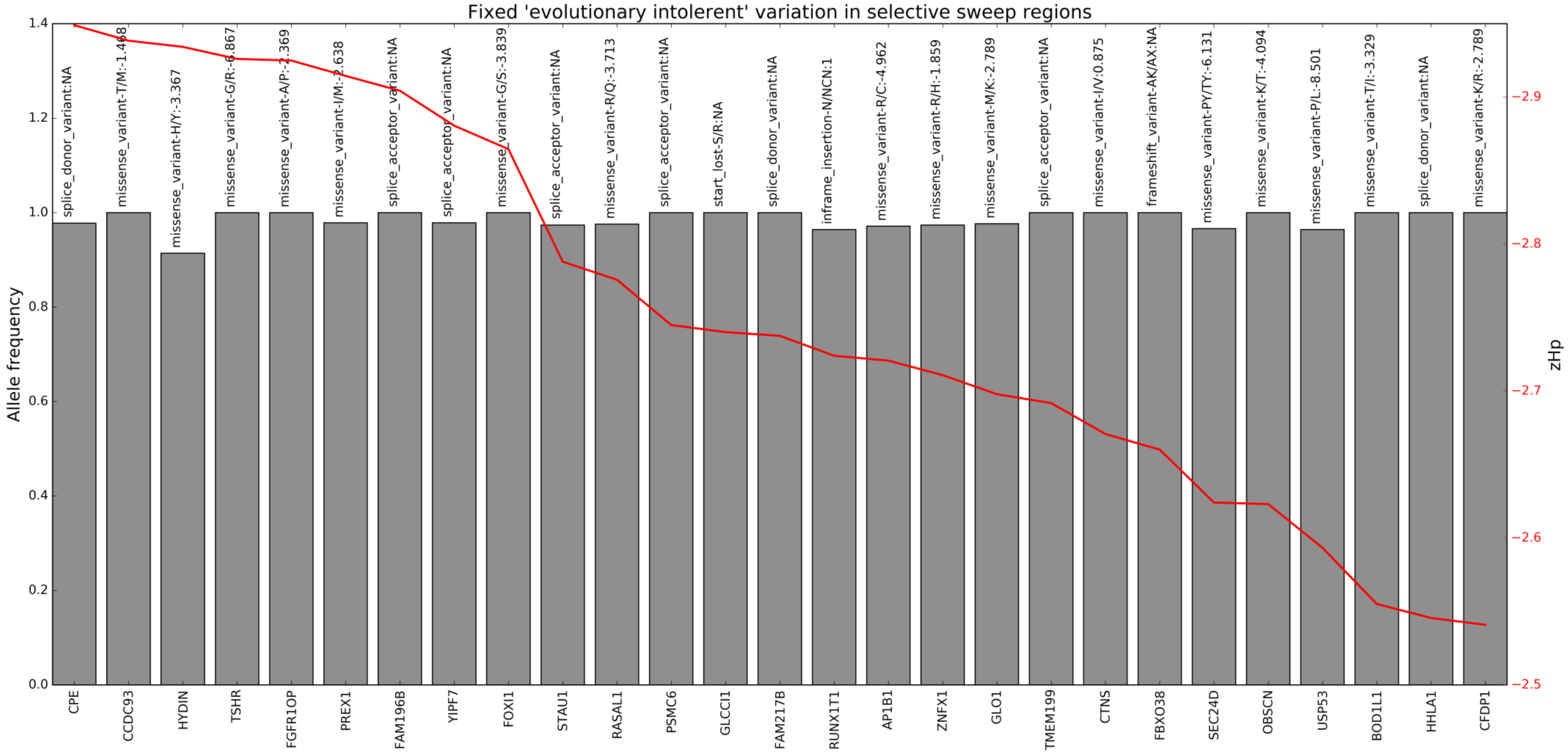

Figure S12: Fixed evolutionary intolerant variants in selective sweep regions. The bars indicate the allele frequency (left y-axis), the red line indicated the zHp score (right y-axis). The genes in which the variant is found is indicated on the x-axis

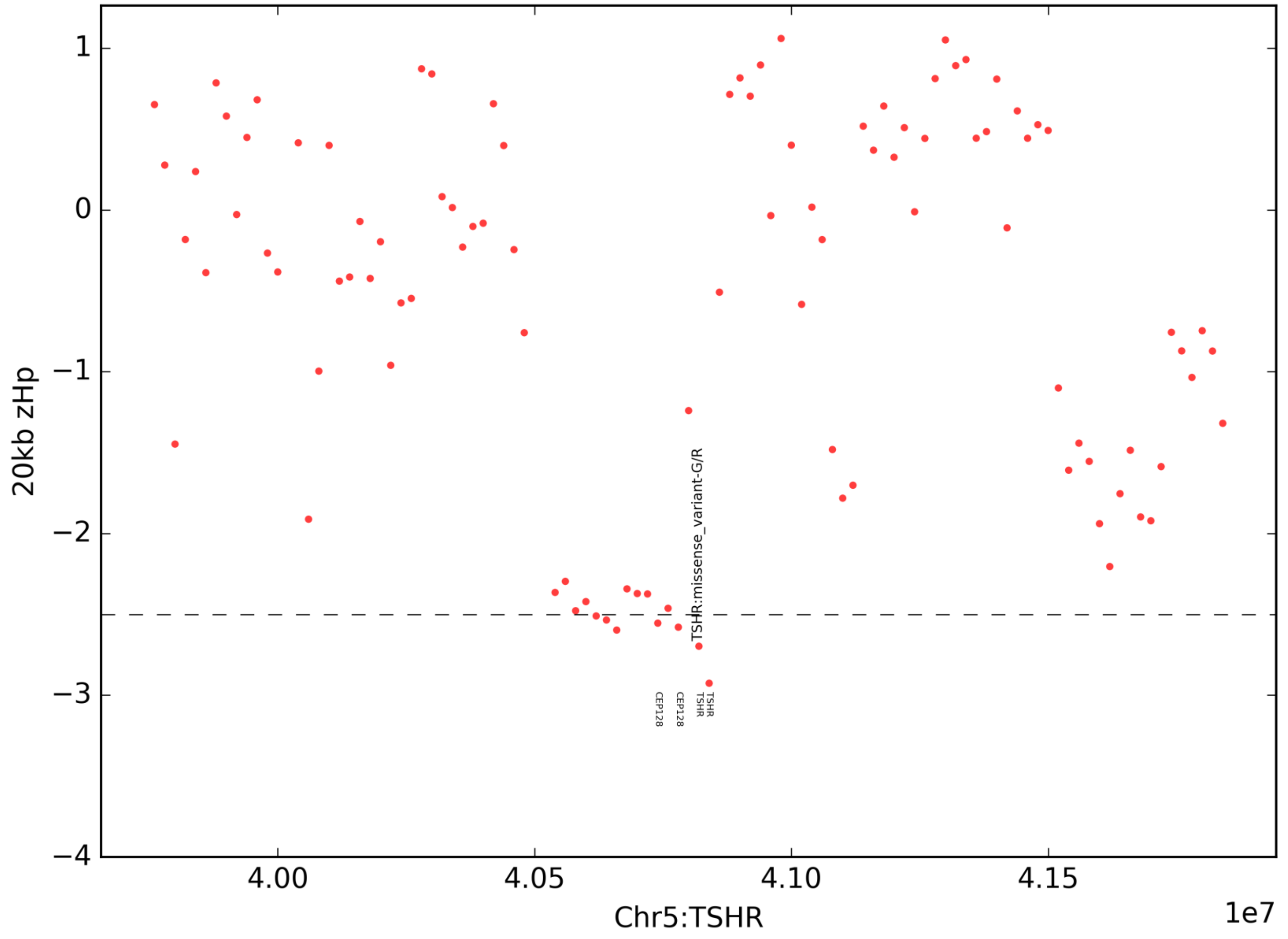

Figure S13: Selective sweep region comprising the TSHR gene including a fixed missense variant (GGA5: 40858336-G>R, AF:100%).

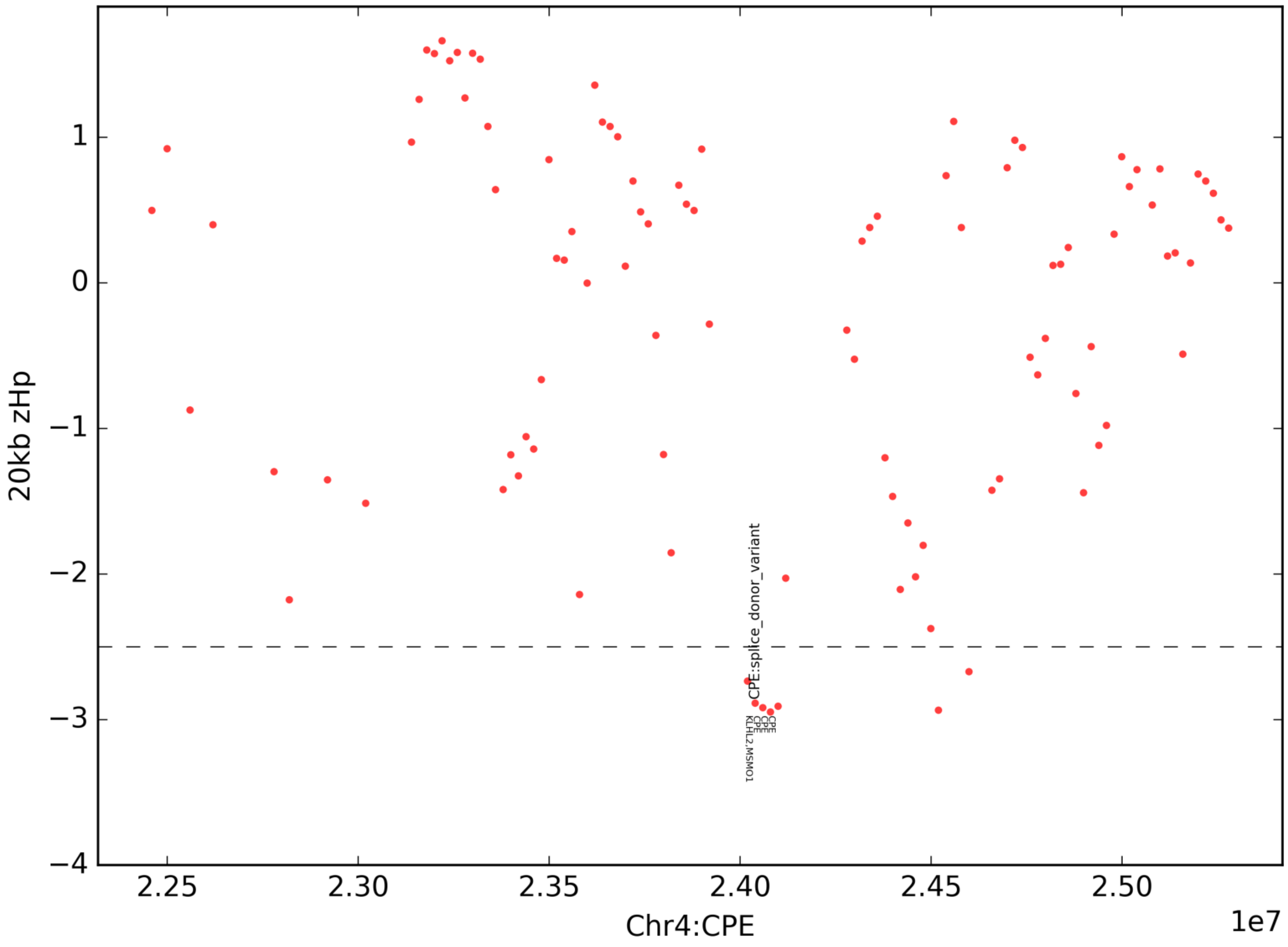

Figure S14: Selective sweep region comprising the CPE gene including a near fixed splice-donor variant (GGA4: 24073667-GG>GGTTTG, AF: 97.8%).

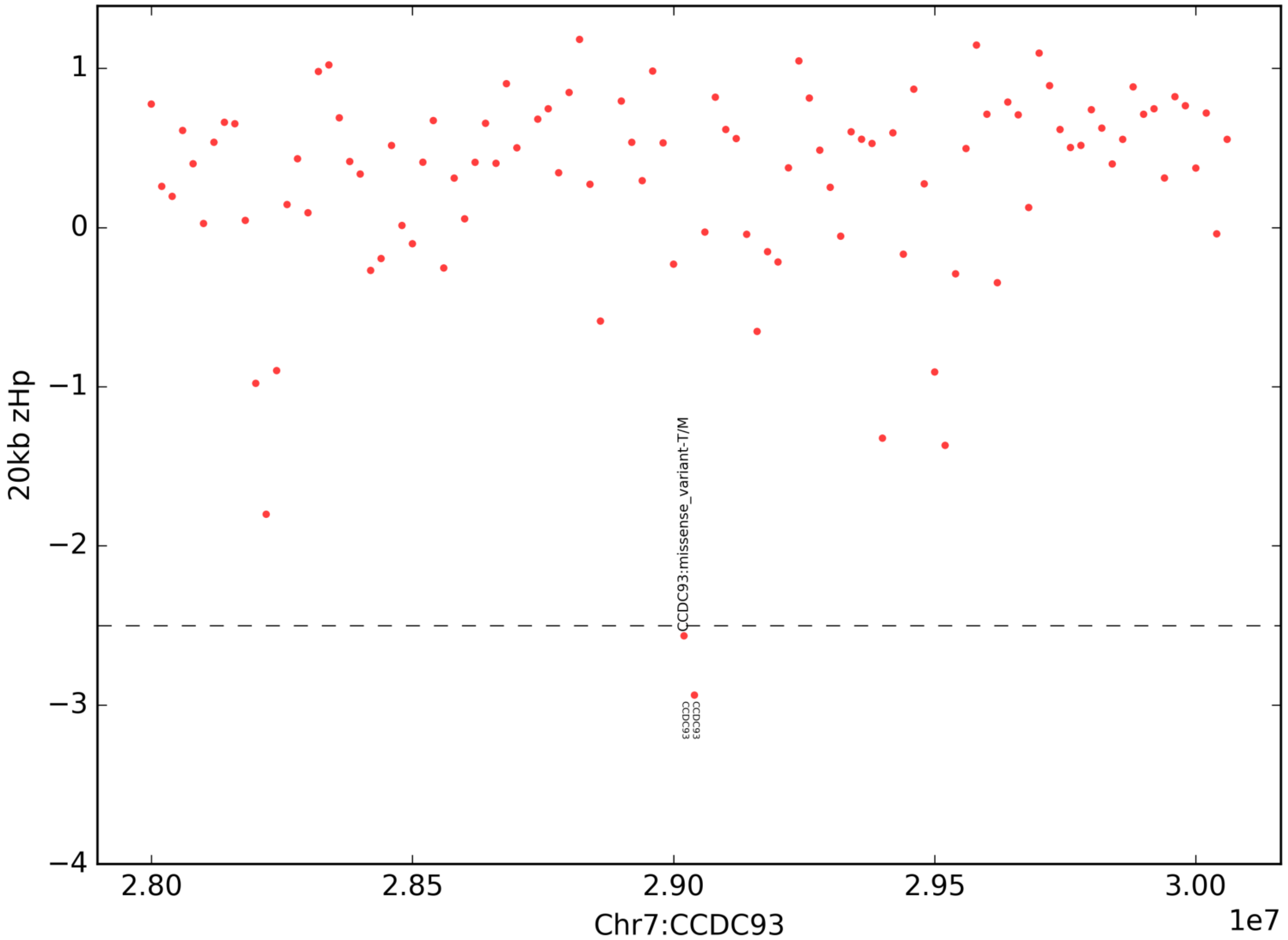

Figure S15: Selective sweep region comprising the CCDC93 gene including a fixed missense variant (GGA7:29050164-T>M, AF: 100%).

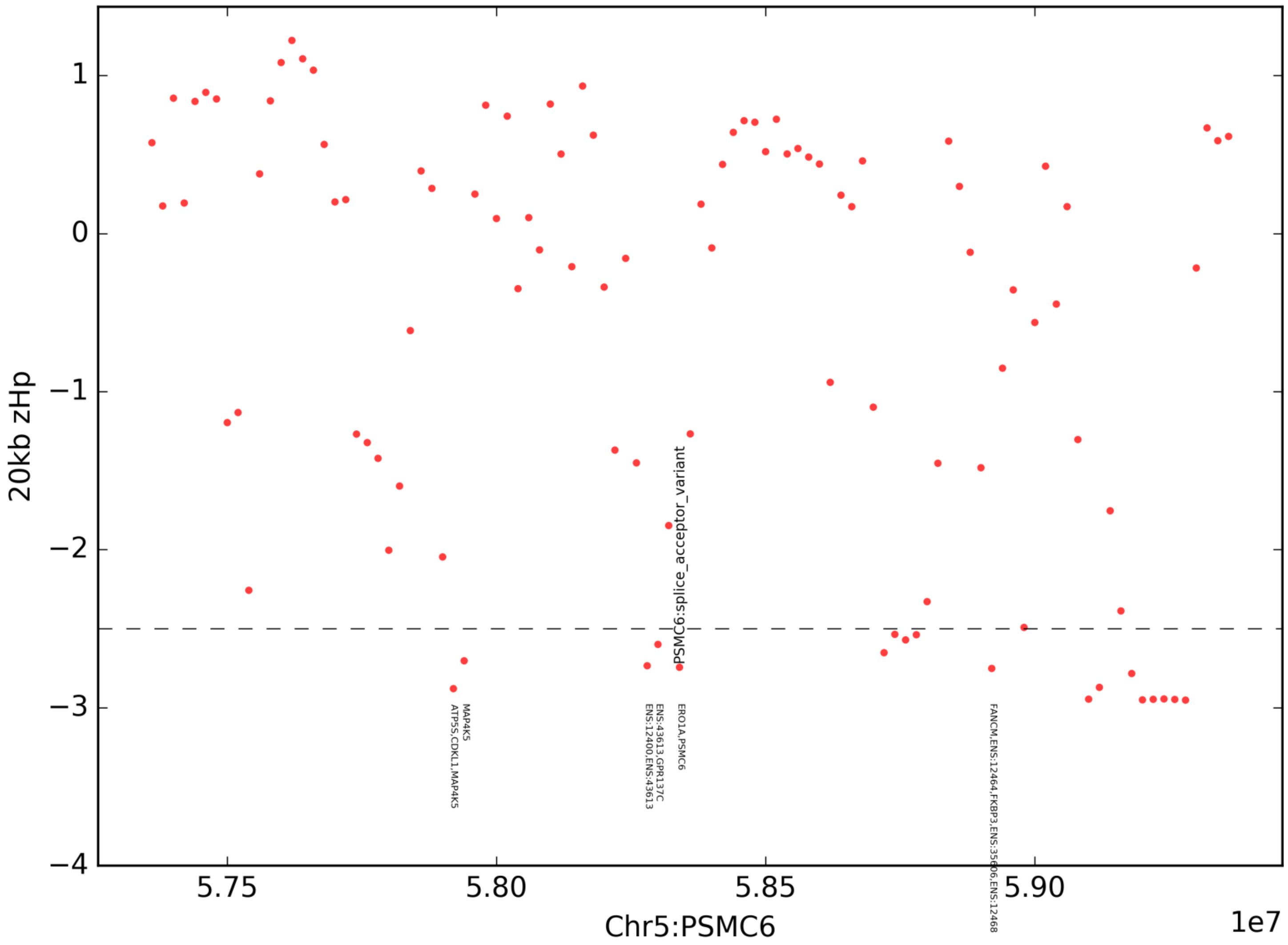

Figure S16: Selective sweep region comprising the PSMC6 gene including a fixed splice-donor variant (GGA5: 58359683-A>C, AF: 100%).

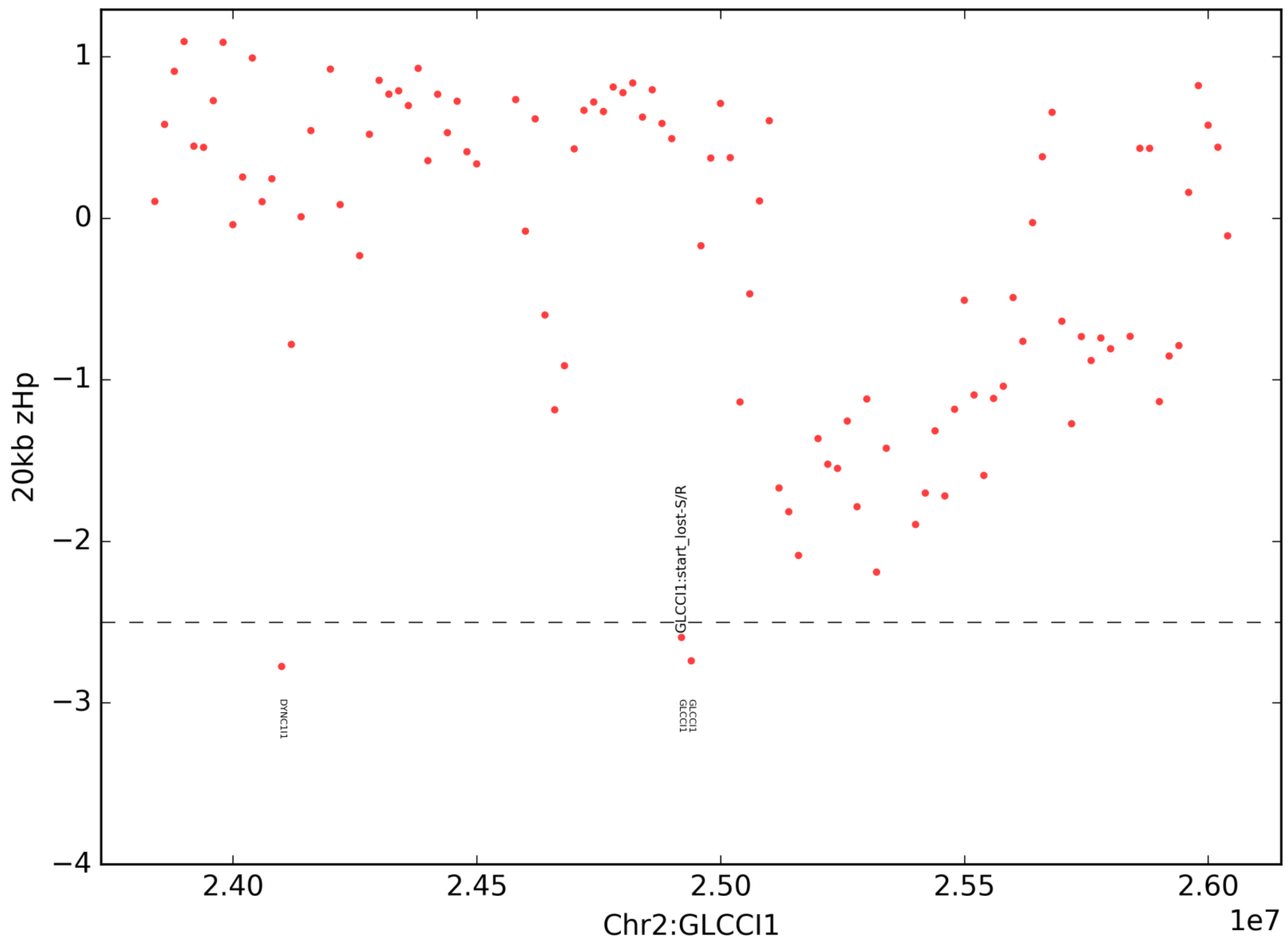

Figure S17: Selective sweep region comprising the GLCC1 gene including a fixed start lost variant (GGA2: 24945757-C>G, AF: 100%).

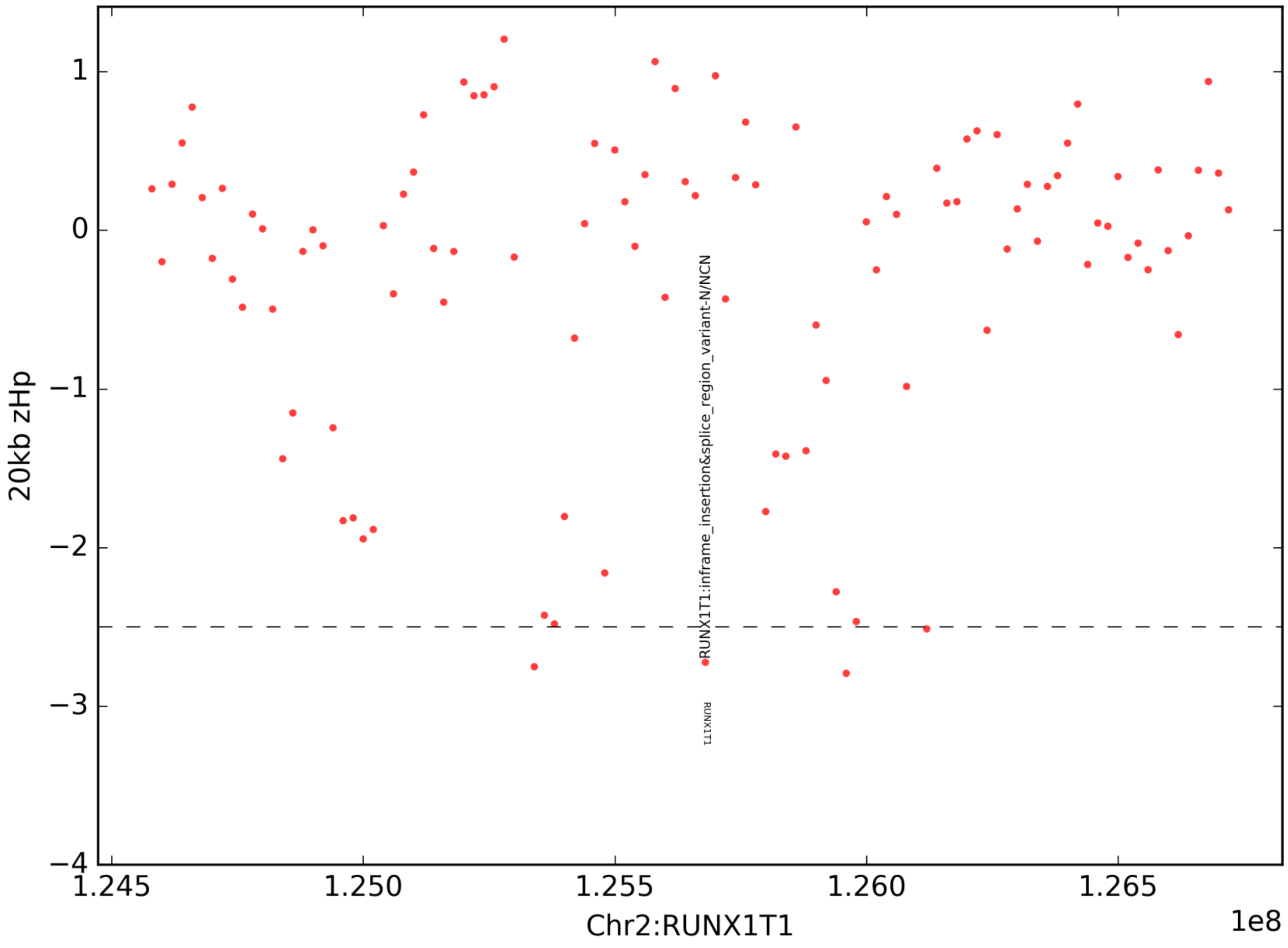

Figure S18: Selective sweep region comprising the RUNX1T1 gene including a near fixed inframe insertion variant (GGA2: 125638723-N>NCN, AF: 96.4%).
